# Modularity and diversity of target selectors in Tn7 transposons

**DOI:** 10.1016/j.molcel.2023.05.013

**Published:** 2023-06-15

**Authors:** Guilhem Faure, Makoto Saito, Sean Benler, Iris Peng, Yuri I. Wolf, Jonathan Strecker, Han Altae-Tran, Edwin Neumann, David Li, Kira S. Makarova, Rhiannon K. Macrae, Eugene V. Koonin, Feng Zhang

**Affiliations:** 1 Howard Hughes Medical Institute, Cambridge, MA 02139, USA; 2 Broad Institute of MIT and Harvard, Cambridge, MA 02142, USA; 3 McGovern Institute for Brain Research, Massachusetts Institute of Technology, Cambridge, MA 02139, USA; 4 Department of Brain and Cognitive Sciences, Massachusetts Institute of Technology, Cambridge, MA 02139, USA; 5 Department of Biological Engineering, Massachusetts Institute of Technology, Cambridge, MA 02139, USA; 6 National Center for Biotechnology Information, National Library of Medicine, Bethesda, MD 20894, USA

**Keywords:** transposon, mobile elements, Tn7, CRISPR, CAST, tyrosine recombinase

## Abstract

To spread, transposons must integrate into target sites without disruption of essential genes while avoiding host defense systems. Tn7-like transposons employ multiple mechanisms for target-site selection, including protein-guided targeting and, in CRISPR-associated transposons (CASTs), RNA-guided targeting. Combining phylogenomic and structural analyses, we conducted a broad survey of target selectors, revealing diverse mechanisms used by Tn7 to recognize target sites, including previously uncharacterized target-selector proteins found in newly discovered transposable elements (TEs). We experimentally characterized a CAST I-D system and a Tn6022-like transposon that uses TnsF, which contains an inactivated tyrosine recombinase domain, to target the *comM* gene. Additionally, we identified a non-Tn7 transposon, Tsy, encoding a homolog of TnsF with an active tyrosine recombinase domain, which we show also inserts into *comM*. Our findings show that Tn7 transposons employ modular architecture and co-opt target selectors from various sources to optimize target selection and drive transposon spread.

## Highlights

• A broad survey of Tn7-like transposons clarifies their target selection strategies
• Type I-D CASTs transpose via both RNA-guided and protein-mediated mechanisms
• Transposon-encoded TnsF is a novel target selector that targets the *comM* gene
• Tsy is a tyrosine recombinase transposon that shares TnsF with Tn7-like Tn6022

## Graphical abstract

**Figure:**
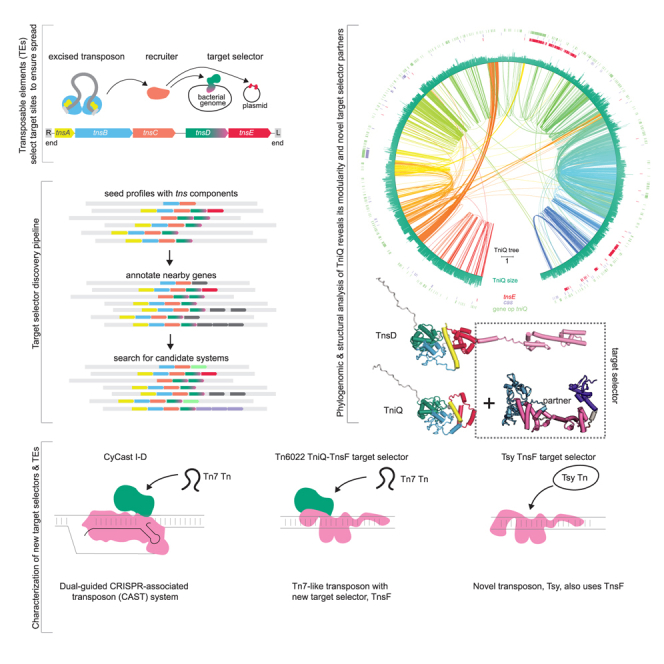

## Teaser

Faure et al. use phylogenomic and structural approaches to systematically survey transposon target selectors, revealing a modular architecture that promotes transposon spread. Through this work, they identified a new target selector, TnsF, and a previously uncharacterized transposable element, Tsy.

## Introduction

**Figure 1:**
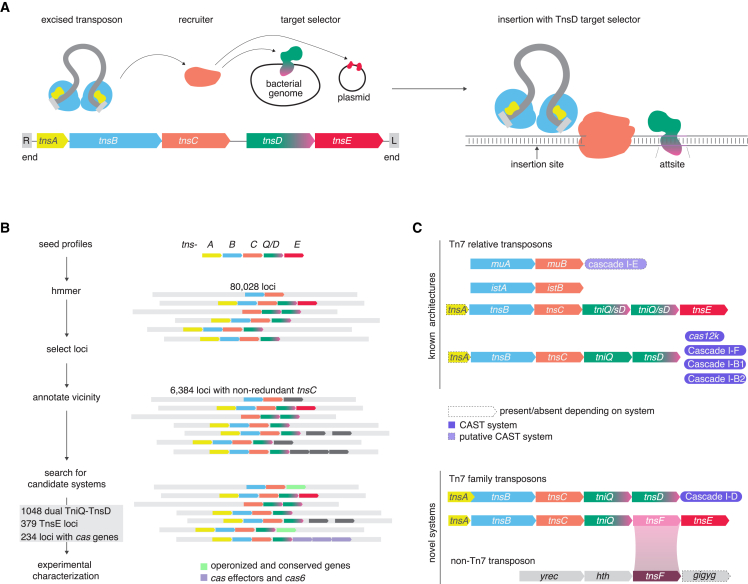
Prediction of novel target selectors in Tn7-like transposons (A) Schematic of Tn7 transposition. TnsB (cyan) recognizes both ends (R, right and L, left) and excises the transposon with the help of TnsA (yellow). TnsD is a sequence-specific target selector and binds an attachment site in the bacterial genome to recruit TnsC (orange) and the transposon for insertion. (B) Pipeline for discovery of novel target selectors. Sequence databases were mined for Tn7 component seeds and searched for genomic co-localization of these seeds. The genomic neighborhoods of the detected loci were annotated, with the focus on *cas* effectors and genes that appear to be operonized with *tnsC* and *tniQ*/*tnsD*. (C) Locus architectures of known systems and novel systems identified in this study. Mu (*muA* and *muB*) and IS21 (*istA* and *istB*) encode relatives of TnsB and TnsC. IS21 has not been reported to be associated with a target selector. Tn7 encodes various target selectors including TniQ/TnsD, TnsE, and Cas effectors (Cas12k, Cascade I-F, and Cascade I-B, which all partner with TniQ), the latter of which constitute CAST systems. We found a novel CAST system containing Cascade I-D and a novel target selector we named TnsF. We also found a TnsF-like target selector in a distinct non-Tn7 transposon.

Transposable elements (TEs) are DNA sequences that can move around and across genomes, employ diverse molecular mechanisms to achieve mobility, and exhibit a broad range of targeting specificities.^1^ Where a TE integrates is critical for its survival, and various strategies have evolved to select target sites, both for homing and for jumping to a mobile genetic element. For example, members of the Tn7 group of prokaryotic DNA transposons recognize (1) a highly conserved sequence of an essential gene to guide integration to a safe locus for homing and (2) a particular DNA conformation (which is agnostic to sequence) to guide integration to mobile elements at replication forks.^2^^,^^3^^,^^4^ These two modes of target recognition are carried out by two dedicated proteins, TnsD (sequence-specific) and TnsE (structure-specific) (Figure 1A). In addition to these two target selectors, Tn7 encodes the heterocomplex TnsA/TnsB, which recognizes the ends of the transposons (TnsB) and excises the transposon (TnsA and TnsB), and TnsC, the central hub component that coordinates the transpososome assembly with target-site selection.^5^ TnsC recognizes TnsD bound to the attachment site, recruits the transpososome, and directs its integration.^6^

In addition to these canonical modes of target-site selection, several groups of Tn7-like transposons have co-opted CRISPR-Cas systems, enabling RNA-guided transposition. These CRISPR-associated transposons (CASTs) target mobile genetic elements (MGEs) using matching spacers encoded in the CRISPR array and Cas-effector components coupled with the small protein TniQ, a homolog of TnsD.^7^^,^^8^^,^^9^^,^^10^^,^^11^^,^^12^ The CASTs target homing sites in two alternative modes, either by RNA-guided transposition or through TnsD, similarly to the canonical Tn7-like transposons.^12^ The CASTs appear to have evolved as a result of the recruitment of CRISPR-Cas effector modules by Tn7-like transposons on multiple independent occasions.^7^^,^^13^ Specifically, different groups of Tn7-like transposons acquired CRISPR subtype I-B (at least twice, independently) and subtype I-F and subtype V-K effectors. In each of these cases, the CRISPR effector module acquired by the TE retained the ability to recognize and bind the target DNA but lost the capacity of typical CRISPR systems to cleave the target DNA. In the case of type I CRISPR effectors, the elimination of the cleavage activity results from the loss of the Cas3 helicase-nuclease, whereas in the case of subtype V-K, it is due to the mutation of the catalytic amino acids in the active site of the RuvC-like nuclease.^8^^,^^9^

The existence of these distinct modes of target-site selection by Tn7-like transposons suggests a high degree of flexibility that maximizes their spread and highlights the utility of multiple, functionally orthogonal target-site selectors. Given this flexibility, comprehensive identification of target selectors is challenging. We used a combination of phylogenomic and structural analyses to discover target selectors. Among these candidate target selectors, we identified and characterized three TE systems: a distinct CAST subtype I-D, a Tn7-like transposon that uses a protein we denoted TnsF as a target selector, and a previously unreported TE we name Tsy. Our results expand the understanding of target selection by Tn7-like transposons, reveal the structural features linked to RNA-guided or protein-guided modes of transposition in CAST systems, characterize the modular architecture of Tn7 target selectors, and discover a distinct target-selector partner co-opted from a previously undescribed non-Tn7 TE.

## Results

### TnsC phylogeny reveals the diversity of target selectors in Tn7-like transposons

**Figure 2:**
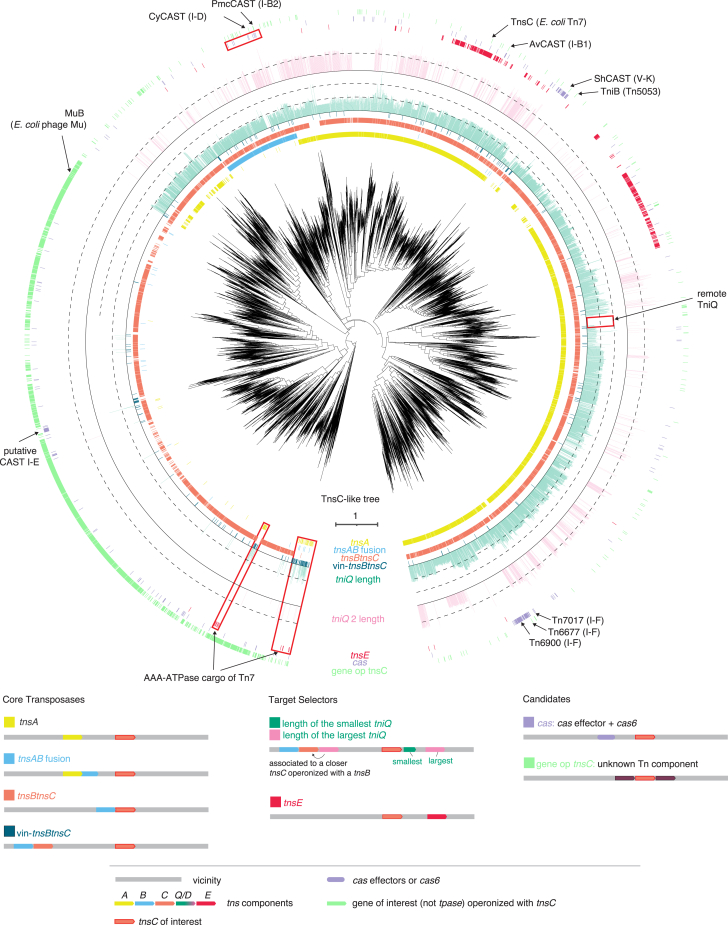
Phylogenetic tree of TnsC homologs Rings around the tree show the presence of a particular gene or a feature in the vicinity of *tnsC* within the genomic contig. From inner to outer ring: *tnsA* is shown in yellow, *tnsAB* fusion is shown in light blue, presence of *tnsB* operonized with the central *tnsC* (representative of the leaf) is shown in orange, presence of an additional distinct *tnsC* operonized with *tnsB* in the vicinity is shown in dark blue, *tniQ/tnsD* is shown in dark green and the presence of a second *tniQ/tnsD* (*tniQ 2*)in pink where both their protein size are proportional to size of the ring bar, *tnsE* is shown in dark red, *cas* effectors and *cas6* genes are shown in purple, the presence of a gene operonized with the central *tnsC* is shown in green. Various known transposons are annotated around the tree including known CAST systems. Red boxes highlight areas of interest. The Mu clade corresponds to the left branch harboring a conserved gene operonized with MuB (homologous to *tnsC*). This gene is part of the Mu phage genome. By subtraction, the Tn7 clade corresponds to the remaining clade and is characterized by the presence of TniQ/TnsD.

Known target-selector proteins exhibit a wide range of diversity, but they all use TnsC to bridge target selection with transposition. We therefore used phylogenomic analysis of TnsC, which is the most prominently conserved protein among the Tn7-encoded proteins, as the framework to investigate the diversity of target selectors and search for new ones (Figures 1B and 1C). We selected 80,028 Tn7-like loci identified in publicly available prokaryotic datasets from NCBI (National Center for Biotechnology Information), JGI (Joint Genome Institute), and MG-RAST (Metagenomic Rapid Annotation using Subsystem Technology), that together included about 1.6 × 10^6^ bacterial and archaeal genomes (STAR Methods) and from these, extracted a representative set of TnsC for phylogenetic analysis. To build the phylogenetic tree, 6,384 TnsC homologs were selected. The tree included two main clades, one consisting of *MuB*—the TnsC homolog from the transposable phage Mu (MuTn)—and the other one of TnsC from Tn7-like transposons (Figure 2).

To explore the diversity of target selectors, we first mapped the known ones (*tniQ/tnsD*, *tnsE*, and *cas* genes from CAST systems) on the tree; *tniQ/tnsD* is ubiquitous in the Tn7 branch and is represented either by a single gene (2,905 loci) or as tandem genes (1,048 loci). These tandems consist of either two *tniQ* genes, or *tniQ* and *tnsD*, or two *tnsD* genes, which we collectively refer to as dual *tniQ-tnsD*. In contrast to the ubiquity of *tniQ/tnsD*, *tnsE* is more restricted in its spread and is present in transposons closely related to the canonical *E. coli* Tn7 and in the more distantly related group of Tn6022 transposons (379 loci total) (Figure 2). CAST systems are spread around the tree and generally grouped according to their subtypes. However, as noted previously, CAST I-B is represented in two distinct clades (1 and 2), suggesting independent capture by two distinct transposons.^12^ We made similar observations for CAST I-F: Tn7017—a CAST I-F variant that harbors a dual *tniQ-tnsD* and uses TnsD for protein-mediated homing^14^—belongs to a branch distant from other CAST I-Fs, which use a dedicated spacer for RNA-guided transposition.^10^ This branch consists of transposons encoding *tnsC* and dual *tniQ-tnsD*, but mostly lacking Cascade I-F, suggesting multiple gains or losses of Cascade I-F.

### Identification of CAST I-D

To identify potential distinct CAST systems, we searched for *cas* genes encoding CRISPR-effector components (see STAR Methods) in the vicinity of *tnsC* and mapped the detected *cas* genes onto the tree (Figures 1 and 2). We identified 234 groups of loci harboring at least one of these *cas* genes. Manual examination of *tnsC* tree branches bearing *cas* genes showed that several of these genes are part of the cargo and are unlikely to be involved in transposition^15^ or belong to already reported CAST systems. However, we identified one group of loci in the Tn7 clade in branches closely related to CAST I-B2 that encodes transposase components closely similar to those of I-B2 PmcCAST, with ∼50% sequence identity between TnsABs and TnsCs and ∼30% sequence identity between the dual TniQ-TnsDs (Figure S1A). However, these loci encoded Cascade I-D, rather than Cascade I-B, and thus comprise a distinct CAST variety.

**Figure 3:**
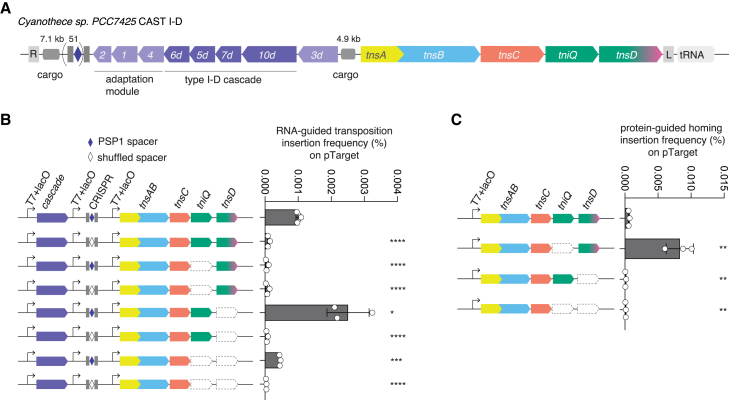
Characterization of CAST I-D (A) Schematic of *Cyanothece* sp. PCC 7425 CAST I-D (CyCAST) locus architecture. (B) RNA-guided insertion frequency of CyCAST into pTarget with PSP1, with or without TniQ and TnsD. (C) Protein-mediated insertion frequency of CyCAST into pTarget with tRNA-leu, with or without TniQ and TnsD. ddPCR experiments were performed with three biological replicates. All data points are shown with an error bar showing standard deviation, and statistical significance was assessed by t test. ^∗^p < 0.05; ^∗∗^p < 0.01; ^∗∗∗^p < 0.001; ^∗∗∗∗^p < 0.0001. See also Figure S1.

To experimentally characterize CAST I-D, we chose a locus from the cyanobacterium *Cyanothece* sp. PCC 7425, CyCAST, which encodes a complete subtype I-D CRISPR-Cas system encompassing both the adaptation module (Cas1, Cas2, and Cas4), the Cas6 processing nuclease, and the Cascade complex, along with TniQ and TnsD (Figure 3A). Unlike other known CASTs, the CRISPR-Cas system of CyCAST appears to be fully functional—that is, competent for both adaptation and interference—based on the conservation of catalytic residues in the HD-nuclease domain of Cas10d, suggesting that this is a recent acquisition of a CRISPR system not yet fully domesticated by Tn7 (Figure S1B). Similar Cas10d proteins are also found encoded in loci where Tn7 components are absent in the vicinity (Figure S1B). Manual identification of the CyCAST boundaries indicated potential attachment sites in a tRNA gene, similar to the attachment site of CAST I-B2 systems (Figure S1A).

We expressed CyCAST heterologously in *E. coli* and tested for activity. Using previously established assays,^8^^,^^9^^,^^12^ we determined that CyCAST exhibits a GTT protospacer-adjacent motif (PAM) preference, as observed in subtype I-D CRISPR-Cas systems^16^ (Figure S1C). We detected mainly unidirectional left-end (LE) and cargo right-end (RE) insertions within a 70 to 80-bp window downstream of the protospacer on a target plasmid (pTarget) (Figure S1D).

To clarify the roles of TniQ and TnsD in RNA-guided insertion, we deleted either TniQ, TnsD, or both and checked for activity. The full CyCAST system with both TniQ and TnsD showed RNA-guided transposition at an insertion frequency of 0.001% (Figure 3B). Deletion of TnsD boosted RNA-guided transposition about 2.5-fold, to 0.0025%, suggesting that TnsD partially inhibits this activity. Elimination of TniQ abolished RNA-guided insertion activity, but perhaps unexpectedly, in the absence of both TniQ and TnsD, a low level of RNA-guided transposition was detected (0.0004%). Thus, CAST I-D retains some basal RNA-guided transposition activity in the absence of a protein-target selector, in contrast to the previously characterized CAST systems, which seems to suggest that TnsC can recognize Cascade at the insertion site (Figure S1E).

We also tested CRISPR-independent transposition (homing) of CyCAST. To this end, we cloned into the pTarget plasmid a leucine tRNA gene from *Cyanothece* sp*.* PCC 7425 to serve as a homing site. Homing transposition was observed when TnsAB, TnsC, and TnsD were expressed in the absence of TniQ (0.008%); the presence of TniQ drastically diminished this activity, but transposition was still detectable (0.0006%) (Figure 3C). Homing transposition occurs around 30–33 nt downstream of the end of the tRNA homing site, resembling the insertion site of CyCAST in the genome of *Cyanothece* sp. PCC 7425 (Figure S1F). Thus, CyCAST exhibits dual modes of transposition that rely on different target selectors, namely, the small TniQ protein for RNA-guided transposition and the larger TnsD for protein-mediated homing.^12^^,^^14^

### Comparison of TniQ and TnsD reveals a modular architecture of target selectors

**Figure 4:**
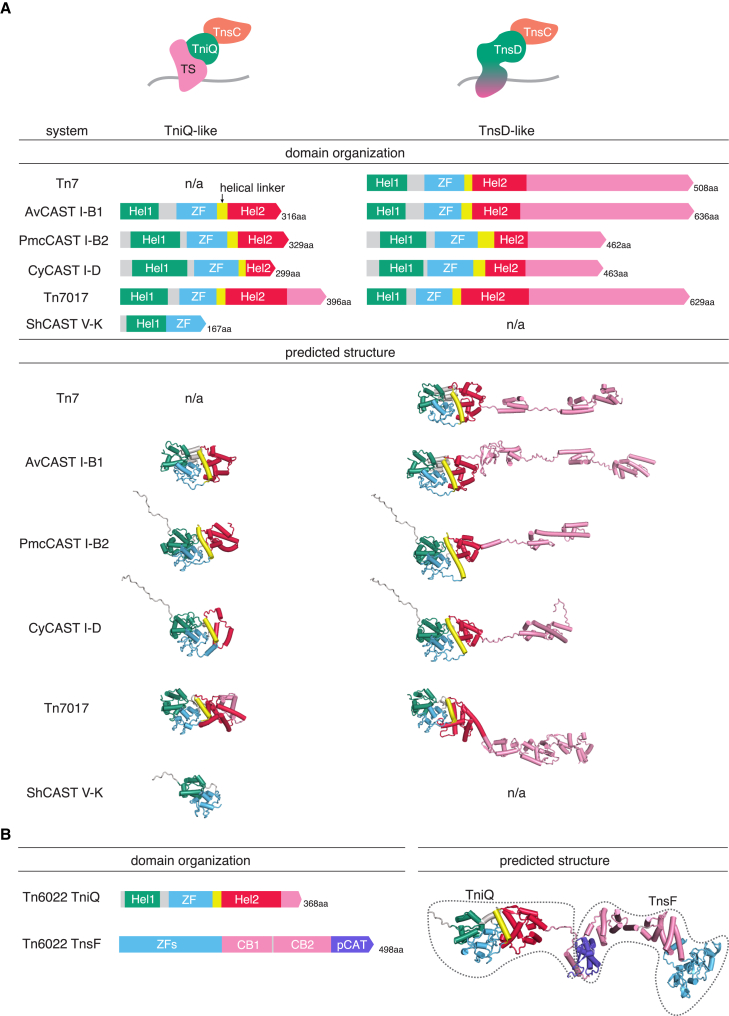
Comparison of dual TniQ-TnsD in CAST systems (A) Domain architecture comparison of TniQ/TnsD. Left: CAST TniQ-like proteins involved in RNA-guided transposition. Right: TnsD-like proteins involved in protein-guided transposition compared with *E. coli* canonical Tn7 TnsD. Except for CAST V-K TniQ, all TniQ/TnsD share a common core region composed of a N-terminal helical domain (Hel1), a zinc finger (ZF), a helical linker, and a C-terminal helical domain (Hel2) (CAST I-D TniQ contains only a partial Hel2 domain). TnsD-like proteins performing protein-guided insertion in CASTs harbor long and diverse C-terminal regions folding into multiple HTH domains similar to Tn7 TnsD. (B) Domain architecture of TniQ and TnsF in Tn6022 (left). Docking prediction of TniQ and TnsF (right). Pink in TniQ indicates C-terminal extension predicted to interact with TnsF. Pink and purple in TnsF indicate the predicted tandem-core binding domain (CB1 and CB2) and the partial catalytic domain (pCAT), respectively. See also Figures S2 and S3.

The presence of dual *tniQ-tnsD* genes in a variety of Tn7 loci motivated us to examine these proteins in greater detail to gain additional insight into their roles in target selection. Mechanistic studies of CAST systems have shown that TniQ, which is smaller than TnsD, partners with Cascade to mediate RNA-guided transposition, whereas TnsD mediates protein-guided transposition (similar to its role in *E. coli* Tn7).^12^ We therefore first focused on TniQ and TnsD from CASTs and non-CAST Tn7-like transposons to determine how they function in these two capacities. We employed structural prediction using AlphaFold2 (AF2)^17^^,^^18^^,^^19^ to compare the domain organizations of TniQ and TnsD variants encoded by *E. coli* Tn7, CAST I-B, CAST I-D, and Tn7017 (the dual TniQ-TnsD CAST I-F) (Figures 4A and S2A). From the structural models, we identified a common core of about 300 amino acids (aa) that consists of a helical domain (Hel1), a zinc finger (ZF), a connector helix, and another helical domain (Hel2). Even within this core region, however, there are notable differences among the TniQ and TnsD proteins from different transposons (Figure S2A). TniQ from Tn6677 CAST I-F interacts with Cas6 as well as Cas7 and the guide RNA through a loop in the Hel2 domain,^20^ suggesting that Hel2 provides a bridge to Cascade and that the structural diversity of Hel2 translates into compatibility with distinct Cascades. By contrast, TniQ from CAST V-K, which associates with the Cas12k effector rather than Cascade, contains only the Hel1 and ZF domains,^21^^,^^22^ indicating a different mode of interaction between the target-selector components and highlighting the flexibility of TniQ as an adaptor between target selection and transposase machineries.

The CAST TniQ proteins consist largely of the core region, whereas the CAST and Tn7 TnsD proteins have diverse, long C-terminal extensions, which might confer target-DNA recognition to enable protein-guided transposition (Figure 4A). However, some of these regions share similarities that might reflect overlapping functions. For example, the TnsDs of Tn7 and CAST I-B, both of which home to the same site (the *glmS* gene),^2^^,^^12^^,^^23^ share common C-terminal domain architectures, which might indicate that this C-terminal region is involved in the recognition of the attachment site. We also detected similar structures and domain architectures between the C-terminal regions of TnsDs from CAST I-B2 and CAST I-D, suggesting that they also recognize similar attachment sites.

### Identification of additional target selectors

The diversity of the interactions between TniQ and Cas effectors raises the possibility that the TniQ core acquired the ability to bind other target-selector proteins as well. Furthermore, these findings suggest that the presence of a small TniQ lacking a large C-terminal extension is a general hallmark of Tn7-like transposons that employ additional target selectors. We therefore expanded our analysis of TniQ and TnsD beyond the CAST systems to systematically analyze their diversity and identify potential partners of TniQ. We extracted the sequences of all TniQ and TnsD proteins encoded in the vicinity of the *tnsC* homologs in the Tn7 clade (5,072 altogether). We found 2,905 loci encoding a single TniQ or TnsD, 998 loci encoding dual TniQ-TnsD proteins, and 50 loci encoding more than 2 TniQ-TnsDs (33 loci with 3, 14 loci with 4, 2 loci with 5, and 1 locus with 6) (Figures 1B and S2B). Loci with more than a single TniQ or TnsD are from Tn7 co-occurring or TniQ-TnsD split into partial genes. TniQ and TnsD protein size distribution falls into four bins, suggesting selection driven by particular size restraints (Figure S2C), but TniQ and TnsD seem to represent two ends of a continuum, spanned by proteins containing extensions of variable length (Figure S2C). This variation makes it difficult to predict, for some of these proteins, whether they bind directly to attachment sites or require a target selector partner. We refer to such proteins as TniQ/TnsD, reflecting this uncertainty.

In dual TniQ-TnsD loci, one of these proteins is usually larger than 400 aa, suggesting direct target selection, whereas the second one is substantially smaller (Figure S2C). Such an architecture could provide transposons with two distinct options for target selection: direct, via TnsD, and indirect, via TniQ interacting with an additional target selector. Analysis of the phylogenetic tree of TniQ/TnsD built using the multiple alignment of the core regions (made from 4,916 proteins passing alignment filters; see STAR Methods) indicates that the dual TniQ-TnsD arrangement is polyphyletic (i.e., it emerged independently on multiple occasions via duplication of a single *tniQ/tnsD* [Figure S2B]), with the monophyly of the dual TniQ-TnsD (i.e., evolution via a single duplication of an ancestral TniQ/TnsD) compellingly ruled out (p value = 2.4e−236). The ancestral Tn7-like transposons likely encoded a single TniQ/TnsD protein, and the dual TniQ-TnsD configurations apparently evolved by *in situ* duplication of a single *tniQ/tnsD* (Figure S2B), followed by neofunctionalization. Such duplication might maintain the compatibility with the other transposase components, while opening the possibility of evolving different modes of target selection. Consistent with the independent duplication scenario, we identified only a few examples of dual TniQ-TnsD loci containing distantly related proteins, as would be expected under an alternative scenario including the exchange of *tniQ*/*tnsD* genes between different transposons (Figures S2B and S2D).

Apart from the dual TniQ-TnsD loci, numerous Tn7-like transposons encompass TniQ together with another, unrelated target selector, such as TnsE, the plasmid-target selector (Figure S2B). We identified two distinct branches encoding TniQ and TnsE (Figures S3A and S3B). Although these TnsEs are highly divergent in sequence (less than 10% of sequence identity), they are predicted to form closely similar structures (Figure S3C). The N-terminal domain of TnsE binds dsRNA via a unique fold,^24^ but the function of this domain has not been explored. Using structural prediction and structural mining^25^ of the N-terminal region, we found that it folds into two single-strand binding (SSB) domains related to PriB (Figure S3D), a component of the bacterial primosome, which can bind both ssDNA and ssRNA and is involved in restarting replication at the fork.^26^^,^^27^^,^^28^ Such structural similarity with PriB suggests that TnsE might have been co-opted from a system that functions at the replication fork. The domain architecture of TnsE has features including a dsDNA-binding domain and a domain predicted to bind ssRNA or DNA, suggesting that it targets the lagging strand of replication. Thus, TnsE is likely to specifically target replication forks of conjugative plasmids, further highlighting the remarkable diversity of target selectors co-opted by Tn7-like transposons.

To search for additional target selectors, we focused on genes operonized with TniQ/TnsD and conserved in multiple nodes of the tree (see STAR Methods and Figure S2D). The most common candidate gene encodes an uncharacterized protein of 498 aa and forms a putative operon with a gene encoding a short TniQ (369 aa) in the Tn6022 family of Tn7-like transposons (121 groups of loci). This gene was previously annotated as orf3 or *tniE*,^29^^,^^30^^,^^31^ but we denote it TnsF (Figure S4A). AF2 prediction of the TnsF structure revealed a distinct domain architecture including an N-terminal region containing multiple ZFs in the first 199 aa, whereas the remaining ∼300 aa exhibit significant structural similarity to the N- and C-terminal domains of the tyrosine recombinase superfamily member XerH (PDB: 5jk0^32^) (Dali score 3.9) (Figures 4B and S4B). However, the chamber holding the tyrosine catalytic site is missing in TnsF (Figure S4B). Tyrosine recombinases typically contain an N-terminal DNA-binding domain (CB domain^32^) and a C-terminal catalytic domain (CAT) and dimerize or tetramerize on DNA during site-specific recombination. Both the N- and C-terminal domains of XerH interact with DNA in the crystal structure (Figure S4B). TnsF is predicted to contain tandem domains (designated CB1 and CB2), which are structurally similar and, by inference, homologous to the CB of XerH (Figure S4B), suggesting that these domains may impart the ability to interact with DNA. A β sheet at the C-terminal region of XerH maps to the last 70 aa of TnsF (designated partial CAT or pCAT) (Figure 4B) and corresponds to the DNA-binding region of the recombinase domain of XerH (Figure S4B) but lacks the helix K.^33^ In XerH, the helix K contributes substantially to its interaction with DNA, suggesting that the pCAT domain of TnsF lost the DNA-binding capacity and might instead interact with TniQ. Indeed, based on an AF2 multimer model, TnsF is predicted to interact with TniQ through its C-terminal region (Figure 4B). Additionally, we found one case where the N-terminal region of TnsF is fused to the C-terminal region of TniQ (GenBank: SCZ64694). This fusion protein lacks the entire CAT but contains an additional CB-like domain within the linker between TniQ and TnsF.

The Tn6022 transposons also encode *tnsE* on the opposite strand, suggesting they can jump to conjugative plasmids, whereas TniQ and TnsF are likely involved in target selection within the bacterial chromosome. Together, these data suggest that TnsF is a distinct target selector and that TniQ of Tn6022 serves as a hub that bridges TnsF, which binds directly to the attachment site, with the transposition machinery.

### TnsF is essential for Tn6022 transposition and interacts with TniQ

**Figure 5:**
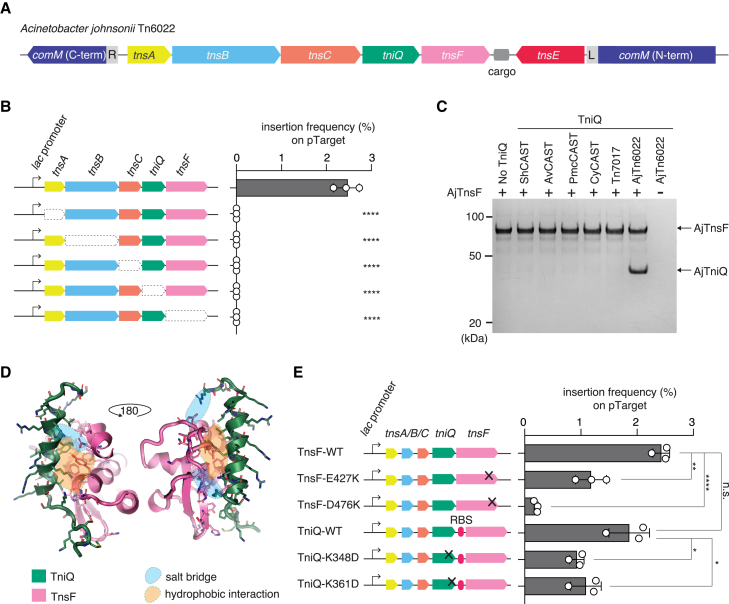
Characterization of TnsF-containing Tn6022 (A) Schematic of *Acinetobacter johnsonii* Tn6022 (AjTn6022) locus architecture. (B) ddPCR experiments showing the insertion frequency of AjTn6022 into pTarget with a 200 bp-fragment of *comM* in the absence of indicated AjTn6022 component. See also Figures S4C and S4D. (C) Protein gel showing TnsF-TniQ interaction. TwinStrep-bdSUMO-TnsF was mixed with the indicated purified TniQ protein, bound to beads, and then eluted complexes were analyzed by gel. Raw gel image and gel showing purified proteins used in the pull-down assay are in SD 2. (D) Molecular details of the interaction region from the docking prediction between Tn6022 TnsF (pink) and TniQ (green). TniQ is predicted to interact via its C-terminal helix with the pCAT domain of TnsF. The interaction involves 2 salt bridges (pale blue areas) and multiple hydrophobic interactions (pale orange areas) (left). (E) ddPCR experiments showing the insertion frequency of AjTn6022 into pTarget with a 200 bp-fragment of *comM* with indicated TnsF and TniQ mutants. For TniQ mutants, overlapped TniQ and TnsF sequences were separated with a ribosome-binding sequence (RBS). ddPCR experiments were performed with three biological replicates. All data points are shown with an error bar showing standard deviation, and statistical significance was assessed by t test; ^∗^p < 0.05; ^∗∗^p < 0.01; ^∗∗∗^p < 0.0001; n.s., not significant.

To experimentally test the predicted target selector function of TnsF, we focused on the Tn6022 transposon. Tn6022 encodes TnsA, TnsB, TnsC, TniQ, TnsF, and TnsE and is inserted in the *comM* gene, which encodes a protein containing a AAA+ ATPase domain and a Mg chelatase domain^34^^,^^35^ (Figure 5A; Data S1). We reconstituted *Acinetobacter johnsonii* Tn6022 (hereafter, AjTn6022) in *E. coli*. We determined the ends of the transposon (see STAR Methods) and cloned the left and right ends into a pDonor plasmid with a kanamycin-resistance gene as a cargo. We also cloned a 100-bp fragment (50 bp upstream and 50 bp downstream of the insertion site) of the *AjcomM* gene into a pTarget plasmid. These plasmids were co-electroporated into *E. coli* with a pHelper plasmid (bearing *tnsA*, *tnsB*, *tnsC, tniQ*, and *tnsF*). To determine the structure of the insertion, we performed long-read, amplification-free nanopore sequencing. We found simple insertions (60.9% of insertions) and co-integrate insertions (39.1% of insertions) (Figure S4C), and we confirmed the presence of target-site duplications (TSDs), a signature of Tn7-like transposition (Figure S4D). To determine whether all Tns proteins including TnsF are essential for transposition into the *comM* gene, we generated pHelper variants lacking each of the Tns proteins and repeated the transposition assay. AjTn6022 achieves transposition at 2.4% efficiency, and removal of any Tns protein (A, B, C, Q, or F) impaired transposition, as quantified by droplet digital PCR (ddPCR) (Figure 5B).

To test our prediction that TnsF and TniQ interact, we purified TnsF and TniQ proteins from AjTn6022, and performed pull-down assays. We showed that *in vitro* TnsF interacts specifically with AjTniQ, but not with TniQ proteins from ShCAST, AvCAST, PmcCAST, CyCAST, or Tn7017 (Figure 5C; Data S2). The docking model of AjTnsF:AjTniQ predicts an interaction between the C-terminal helix of TniQ and the pCAT region of TnsF via hydrophobic contacts and salt bridges (Figure 5D). TniQ from other systems lack this helical region, which may explain why they do not interact with AjTnsF. Mutants designed to disrupt the predicted salt bridges (TnsF E427K/D476K and TniQ K348D/K361D) abrogated or substantially reduced transposition (Figure 5D), supporting the hypothesis that this Aj-specific region of TniQ is important for the interaction with TnsF.

### Identification of Tsy, a non-Tn7 transposon that uses TnsF for target selection

**Figure 6:**
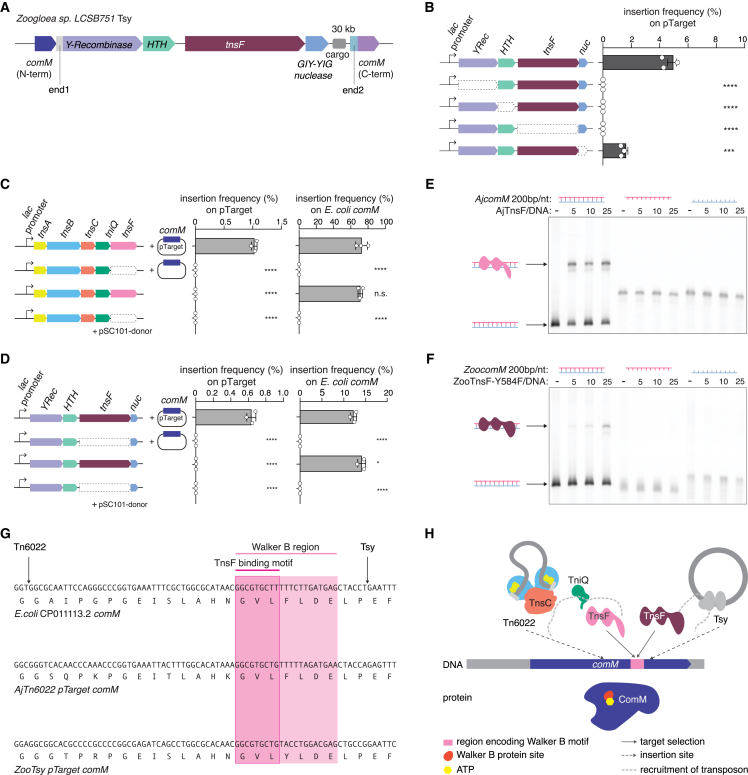
TnsF targets a conserved Walker B motif in *comM* (A) Schematic of the locus architecture of *Zoogloea* sp. *LCSB751* target selector based on tyrosine (Y) recombinase transposon (ZooTsy). (B) Genetic requirement of YRec, HTH, and TnsF on ZooTsy transposition activity, as assayed by quantification of upstream-end1 junction formation by ddPCR. Deleted genes are indicated by a dashed outline. See also Figure S5H. (C) ddPCR experiments showing the insertion frequency of AjTn6022 into pTarget with a 200 bp-fragment of *comM* (left) and *E. coli* endogenous *comM* (right) in the absence of TnsF and/or presence of the pTarget(AjTn6022). pSC101 donor was used. (D) ddPCR experiments showing the insertion frequency of ZooTsy into pTarget with a 200 bp-fragment of *comM* (left) and *E. coli* endogenous *comM* (right) in the absence of TnsF and/or presence of the pTarget(ZooTsy). pSC101 donor was used. (E) Electrophoretic mobility shift assay (EMSA) to assess the interaction between a 200-bp or 200-nt fragment of AjcomM and purified AjTn6022-TnsF. (F) EMSA to assess the interaction between a 200-bp or 200-nt fragment of ZoocomM and purified ZooTsy-TnsF_Y584F. (G) Insertion sites of Tn6022 and ZooTsy on *E. coli* endogenous *comM* and *comM* of their respective hosts used in the pTarget. ComM protein sequences (translated *comM*) are shown below the nucleotide sequence. The pink rectangle indicates the genomic location of the Walker B of the AAA ATPase encoded by *comM*; the red rectangle shows the probable hot spot binding region of both TnsFs. (H) Model of TnsF target selection and insertions for Tn6022 and Tsy. ddPCR experiments were performed with three biological replicates. All data points are shown with an error bar showing standard deviation, and statistical significance was assessed by t test. ^∗^p < 0.05; ^∗∗∗^p < 0.001; ^∗∗∗∗^p < 0.0001; n.s., not significant. See also Figures S5–S7.

We next searched for TnsF homologs in genomic databases and identified 1,099 nonredundant TnsF homologs (STAR Methods; Data S1), including Tn6022 TnsF and a homolog of TnsF (referred to as TnsF-like protein) containing a predicted active CAT (Figures S4A and S4B). Although we also detected more distant structural homologs of TnsF containing adjacent CB and CAT domains, these proteins lacked the ZF-containing N-terminal regions, so we did not include them in the further analysis (Figure S4E). We built a phylogenetic tree of the TnsF and TnsF-like proteins (Figure S4F) and mapped on it the conserved genes located in the vicinity of *tnsF* and *tnsF*-like genes. Tn7 TnsF forms a distinct clade and apparently evolved from TnsF-like proteins with an active tyrosine recombinase CAT. The genomic neighborhood of these TnsF-like proteins lacks Tn7 components but instead includes upstream genes encoding a tyrosine recombinase (YRec) and a small helix turn helix (HTH)-domain protein as well as a downstream gene encoding a GIY-YIG nuclease (present only in the branch close to Tn7) (Figures 6A and S4F). Although we could not detect inverted repeats or any canonical ends in these loci, we noticed the presence of *comM* fragments, namely, the 5′ terminal portion located upstream of *YRec* and the 3′ terminal portion located downstream of the putative transposon (Figure 6A). The downstream portion of the *comM* gene is in some cases located after several additional genes, which likely represent transposon cargo. These features suggest that this locus is a distinct transposon and that the TnsF-like protein recognizes *comM*, similarly to TnsF of Tn6022.

To determine the function of these potentially enzymatically active TnsF-like proteins, we experimentally characterized this mobile element, which we designate Tsy (target selector based on tyrosine recombinase). We reconstituted the system from *Zoogloea* sp. *LCSB751* (hereafter, ZooTsy) in *E. coli*. To assess ZooTsy transposition, we cloned 135 and 39 bp of each transposon end1 (*comM* 5′ terminal portion) and end2 (*comM* 3′ terminal portion) with 12 bp of homology arm extensions into an R6K origin pDonor plasmid with a kanamycin-resistance gene as cargo (Figures S5A and S5B). We also cloned a Tsy attachment site (a 100-bp fragment with 50 bp upstream and 50 bp downstream of the *Zoogloea* sp. *comM* insertion site) into a pTarget plasmid (Figure S5A and S5B). These plasmids were co-electroporated into *E. coli* with a pHelper plasmid (bearing *YRec*, *HTH*, *tnsF*, and the GIY-YIG nuclease, *nuc*). We detected transposition into pTarget and observed circular intermediates (CIs) derived from pDonor by PCR in a YRec-dependent manner (Figure S5A), as previously demonstrated for various transposons encoding tyrosine recombinases.^34^ To confirm these findings, we established an assay to isolate and confirm the structure of the CI derived from the pDonor. We constructed a derivative pDonor that contained the ColE1 origin of replication and kanamycin resistance gene as cargo as well as lacZα but no other origin of replication. Upon circularization, this pDonor will lose lacZα and can be isolated from white colonies by traditional blue-white screening (Figure S5A). Using this assay, we obtained white colonies (98% of the total) in a pHelper-dependent manner after retransformation with the extracted plasmids and successfully isolated a smaller plasmid which had lost the 0.6-kb backbone region of the pDonor (Figure S5A; Data S3). We confirmed the smaller plasmid as a CI by nanopore long-read sequencing and observed the connected end2 (…AATCCCAGTC) and end1 (AAGTTCTGAT…) junction by Sanger sequencing (Figure S5A; Data S3). To determine the structure of the ZooTsy insertions, we performed nanopore long-read sequencing and found simple insertions (62.3% of total insertions) (Figures S5C and S5D).

To narrow down the requirements for transposition, we generated variants of the cargo with serial deletions of end1 from 135 to 0 bp, finding that truncation of this end gradually decreased the rate of simple insertions to zero (Figure S5E). By contrast, only 20 bp at end2 are required for transposition (Figure S5E). By systematically combining these optimized parameters, we found that 12 bp of homology arm 1 (hom1), 135-bp end1, and 20-bp end2 are sufficient for the transposition of ZooTsy (Figure S5F).

To determine the genetic requirements for ZooTsy transpositions in *E. coli*, we constructed a series of pHelper plasmids with deletions of each gene. ZooTsy achieves transposition at 5.0% efficiency, and removal of any component (YRec, HTH, TnsF, or GIY-YIG nuclease) from the system impaired transposition, as quantified by ddPCR for the upstream-end1 junction formation (Figures 6B and S5G). The GIY-YIG nuclease, however, is not essential for transposition, which is supported by the identification of Tsy relatives lacking this component (Figure S4E). By contrast, we found that the tyrosine recombinase catalytic activities of both YRec and TnsF are essential for transposition (Figure S5H).

### TnsF targets a conserved region of *comM*

Both AjTn6022 and ZooTsy use TnsF to target *comM* of their respective hosts, although the directionality of insertion is different, suggesting different modes of insertion. ComM has been reported to facilitate recombination of sequences acquired by transformation,^35^ and disruption of the *comM* gene by transposon insertion could inactivate this functionality, limiting transformation of other MGEs. To explore *comM* targeting in greater depth, we sought to compare TnsF-mediated targeting of this gene by AjTnsF and ZooTnsF. Using our heterologous *E. coli* expression systems, we looked for AjTn6022 and ZooTsy targeting of genomic *comM*, finding that they can both target *E. coli* endogenous *comM* in addition to their respective *comM* sites on pTarget, highlighting the broad recognition of *comM* by both TnsFs (Figures 6C and 6D). To determine the specificity of *comM* gene targeting by TnsF, we performed tagmentation-based tag integration site sequencing (TTISS).^36^ For AjTn6022, in the absence of pTarget, we observed that 96.7% of insertions were at the *E. coli* endogenous *comM* locus; in the presence of pTarget, 56.6% of insertions were on pTarget and 40.8% were on the genomic *comM* (97.4% on target in total) (Figure S6A). For ZooTsy, we observed similarly high levels of specificity, indicating that TnsF is highly selective for *comM* (Figure S6B). We confirmed that both TnsF proteins bind 200-bp dsDNA fragments corresponding to their respective *comM* target sequences (Figures 6E and 6F).

To further characterize the attachment sites of these proteins, we constructed additional pTarget variants with different lengths of *comM* gene fragments to map the target-site specificity of both TnsFs at a greater resolution. For AjTn6022, deletion of the 40–50 bp of either upstream or downstream sequences of the insertion site substantially reduced transposition, indicating that the insertion site was located within the TnsF attachment site (Figure S6C). For Tsy, we found the attachment site is within a 40-bp upstream region from the insertion site (Figure S6D). Mapping these refined target sites on the respective *comM* genes showed that they overlap with a conserved 10-bp region within the Walker B motif (a highly conserved ATPase motif^37^) of the ComM protein (Figure 6G). We confirmed that this 10-bp region is necessary for both AjTnsF and ZooTnsF binding (Figures S7A and S7B). Furthermore, we found that mutating this region abolished the transposition activity of both AjTn6022 and ZooTsy (Figures S7C and S7D). Targeting the Walker B motif of *comM* might provide a natural conserved anchor for TEs to spread across species, paralleling Tn7 targeting of the catalytic site in *glmS*. Together, these results demonstrate that TnsF is a target selector related to the tyrosine recombinase family and involved in the transposition of at least two distinct groups of transposons.

## Discussion

**Figure 7:**
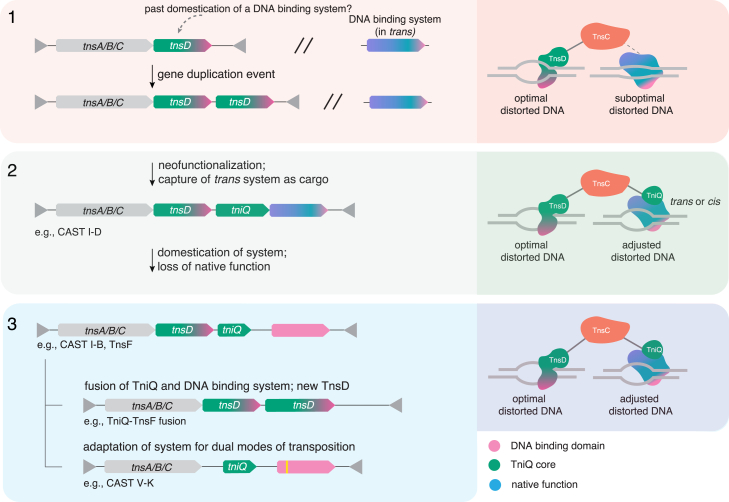
Evolution of the functional versatility of TniQ-interacting target selectors Evolutionary scenarios for various Tn7-like transposons with distinct modes of target selection. Locus architecture is shown on the left and mechanics of target-site selection on the right. (1) An ancestral Tn7-like transposon might have used TnsD for site-specific target selection and a DNA-bending protein or complex (e.g., Cas effector, transcription factor, or tyrosine recombinase) in *trans* as a second mode of target-site selection. These DNA-bending proteins would create a distortion in the DNA that TnsC would recognize, albeit with a low efficiency. Gene duplication produced a second copy of TnsD. (2) Neofunctionalization of the second copy of TnsD yielded TniQ, which evolved to optimize the interaction between a *trans* DNA-bending target selector and the target site. The *trans* system could also be captured by the transposon as cargo, as was the case with CRISPR-Cas systems. (3) Further domestication of the target selectors would then occur, eventually leading to the loss of the native function of the system (e.g., CASTs I-B and I-F), fusion to TniQ generating a distinct TnsD (e.g., TniQ-TnsF fusion), or adaptation of the system for dual modes of transposition as in CAST V-K, which relies entirely on the CRISPR system for both homing and jumping. Pink indicates DNA-binding function; green indicates TniQ core; blue indicates native function of DNA-binding system.

Target-site selection is a crucial step in the life cycle of TEs because insertion of the element can dramatically affect the fitness of both the host and the TE depending on the insertion site.^38^^,^^39^ Moreover, the choice of target site is critical for the TE to spread horizontally. Here, we expand the current understanding of target-site selection mechanisms, identifying previously uncharacterized target-site selectors and a distinct family of TE. Together, our results reveal the modular architecture used by Tn7-like transposons to bridge target-site selection with transposase activity, providing TEs with maximal targeting flexibility (Figure 7).

This flexibility is manifested in the various proteins that Tn7-like transposons have co-opted and adopted for target-site selection. Although the CRISPR systems in most CASTs have lost their ancestral interference and nuclease functions, the CyCAST system we describe here has an interference component and a Cas10d protein with an active HD-nuclease domain. Very recently, other CAST I-D systems have been reported—but these lack Cas3d, and Cas10d is inactivated.^40^ Thus, CyCAST seems to represent an evolutionary intermediate where the CRISPR system has been co-opted by the TE but has not fully lost its native function. Along similar lines, we found that the Tn7-like transposon Tn6022 has co-opted TnsF, a catalytically inactive derivative of a tyrosine-recombinase-containing protein, whereas the Tsy transposon we describe here encodes the apparent ancestor of TnsF, a catalytically active recombinase.

Our finding that additional enzymes have been co-opted as target selectors by Tn7-like transposons raises the question of how these recruited proteins evolve target-site selection capacity. In Tn7, to recruit the transposase machinery to the target site, TnsD binds to and induces a local distortion in the target DNA, which then attracts TnsC. Artificial induction of such DNA distortion has been shown to attract TnsC independently of TnsD.^23^^,^^41^ Native Cas effectors^42^ and TnsF likely induce similar distortions in the target DNA, through R-loop formation by Cas effectors and DNA bending by tyrosine-recombinase-containing proteins such as TnsF. Although a canonical tyrosine recombinase would entirely cover the distorted DNA, precluding TnsC access, the distinct architecture of TnsF, with two different DNA-binding domains, might bend the target DNA while still allowing partial or full access for TnsC. Thus, proteins that provide more efficient target selection could supersede the target-selecting role of the C-terminal region of TnsD, ultimately leading to their domestication and loss of their native enzymatic activity (Figure 7). CAST-I-D and the TnsF homolog in the Tsy system are examples of apparent intermediate stages on the evolutionary path to domestication. We also detected numerous loci encoding a TniQ that is too short to enable target selection and no other identifiable target-selector partner. Such elements might recruit target selectors in *trans*, perhaps representing the initial step in the evolution of new target selectors or an even greater flexibility in target-selector recruitment.

### Limitations of the study

Given the apparent fast evolution of target-selector proteins, our sequence-based mining might have limited the scope of our analyses. The recent advances in protein-structure prediction now enable structure-based mining, which could yield candidates beyond those reported here. Indeed, although a sequence-based search did not identify TnsF homologs in other systems, a structural-mining approach might shed light on the potential origins of target selectors, as exemplified by the relationship between TnsE and PriB (Figure S3). These types of evolutionary and structural analyses, combined with further study of the mechanisms of TnsF and Tsy, will shed light on this distinct mode of target-site selection. The identification of distinct target selectors described here highlights the remarkable plasticity of the insertion machinery of Tn7-like transposons, but further research will likely reveal additional mechanisms of transposon targeting.

## STAR★Methods

### Key resources table

**Table:** 

REAGENT or RESOURCE	SOURCE	IDENTIFIER
**Bacterial strains**		

One Shot™ PIR1 Chemically Competent E. coli	ThermoFischer	C101010
BL21(DE3) Competent E. coli	New England Biolabs	C2527
BL21(DE3) Electrocompetent Cells	Millipore Sigma	CMC0016
Endura™ ElectroCompetent Cells	Lucigen	60242
One Shot™ Stbl3™ Chemically Competent E. coli	ThermoFischer	C737303
NEB® 10-beta Competent E.coli (High Efficiency)	New England Biolabs	C3019

**Chemicals, peptides, and recombinant proteins**		

NEBNext® High-Fidelity 2X PCR Master Mix	New England Biolabs	M0541
Q5® High-Fidelity 2X Master Mix	New England Biolabs	M0492
KAPA HiFi HotStart ReadyMix	Roche	KK2602
KOD Hot Start DNA Polymerase	Millipore Sigma	71086-3
E-Gel™ EX Agarose Gels, 1%	ThermoFischer	G401001
E-Gel™ EX Agarose Gels, 2%	ThermoFischer	G401002
Novex™ TBE Gels, 6%, 15 well	ThermoFischer	EC62655
Novex™ Hi-Density TBE Sample Buffer (5X)	ThermoFischer	LC6678
SYBR™ Gold Nucleic Acid Gel Stain (10,000X Concentrate in DMSO)	ThermoFischer	S11494
Wizard® SV Gel and PCR Clean-Up System	Promega	A9282
Wizard® Genomic DNA Purification Kit	Promega	A1120
QIAquick PCR Purification Kit	Qiagen	28106
QIAprep Spin Miniprep Kit	Qiagen	27106
PureYield™ Plasmid Midiprep System	Promega	A2495
Gibson Assembly® Master Mix	New England Biolabs	E2611
NEBuilder® HiFi DNA Assembly Master Mix	New England Biolabs	E2621
Q5® Site-Directed Mutagenesis Kit	New England Biolabs	E0554
BstZ17I-HF®	New England Biolabs	R3594
NruI	New England Biolabs	R0192
BsaI-HF®v2	New England Biolabs	R3733
BbsI-HF®	New England Biolabs	R3539
AarI	ThermoFischer	ER1581
T4 DNA Ligase	New England Biolabs	M0202
IPTG	Goldbio	I2481C
S-Gal®/LB Agar Blend	Millipore sigma	C4478
ddPCR Supermix for Probes (No dUTP)	Bio-Rad	#1863024
Droplet Generation Oil for Probes	Bio-Rad	#1863005
NEBNext® Companion Module for Oxford Nanopore Technologies® Ligation Sequencing	New England Biolabs	E7180S
Ligation Sequencing Kit	Oxford Nanopore Technologies	SQK-LSK109
Flow Cell (R9.4)	Oxford Nanopore Technologies	FLO-MIN106
AMPure XP for PCR Purification	Beckman Coulter	A63881
Tn5	Schmid-Burgk et al.^36^	N/A
cOmplete Protease Inhibitor Cocktail	Millipore sigma	4693116001
Strep-Tactin® Sephaarose® Resin	IBA	2-1201-002
Strep-Tactin® Magnetic Microbeads	IBA	6-5510-050
D-Desthiobiotin	Millipore sigma	71610-3
Ulp1 SUMO protease	F. Zhang Lab	N/A
SENP protease	F. Zhang Lab	N/A
Amicon Ultra-15 Centrifugal Filter Units 10kDa NMWL	Millipore sigma	UFC901024
Amicon Ultra-15 Centrifugal Filter Units 50kDa NMWL	Millipore sigma	UFC905024
NuPAGE™ 4–12% Bis-Tris Protein Gels, 1.0 mm, 12-well	ThermoFischer	NP0322BOX
NuPAGE™ LDS Sample Buffer (4X)	ThermoFischer	NP0007
Imperial™ Protein Stain	ThermoFischer	24615
Ampicillin, sodium salt	AmericanBio	Ab00115
Carbenicillin disodium salt, 89.0–100.5% anhydrous basis	Millipore sigma	C1389
Spectinomycin dihydrochloride pentahydrate	Millipore sigma	S4014
Kanamycin sulfate from Streptomyces kanamyceticus	Sigma	K4000
Chloramphenicol	Sigma	C0378
MiSeq Reagent Kits v2	Illumina	MS-102
NextSeq 500/550 High Output Kit v2, 75 cycles	Illumina	FC-404-2005

**Critical commercial assays**		

Qubit 1X dsDNA HS (High-Sensitivity) Assay Kit	ThermoFischer	Q33231
eStain L1 Protein Staining System	GenScript	N/A

**Deposited data**		

Deep sequencing data	SRA	SRA: PRJNA913200
InsertionReadsCounter	Zenodo	10.5281/zenodo.7872374

**Oligonucleotides**		

CTTTCCCTACACGACGCTCTTCCG ATCTgagcaagagattacgcgcagac	Genewiz	NGS pTarget 6N-upstream primer
CTTTCCCTACACGACGCTCTTCCG ATCTctaccgcattaaagcttccgcc	Genewiz	NGS pTarget 6N-downstream primer
GACTGGAGTTCAGACGTGTGCTC TTCCGATCTgcctattgctttcgctctatctgtcc	Genewiz	NGS pDonor(CyCAST)-LE primer
GACTGGAGTTCAGACGTGTGCTCTT CCGATCTcctaaggcaacacaacggctg	Genewiz	NGS pDonor(CyCAST)-RE primer
CTTTCCCTACACGACGCTCTTCCG ATCTgtaagccttgcgagttcgattctcg	Genewiz	NGS tRNA-leu primer
ctaccgcattaaagcttccgcc	Genewiz	pTarget ddPCR 6N-downstream primer
cctaaggcaacacaacggctg	Genewiz	pDonor(CyCAST) ddPCR for RE primer
cctggtgtccctgttgataccg	Genewiz	pDonor(CyCAST) ddPCR tRNA-leu downstream primer
cgacagcatcgccagtcactatg	Genewiz	pTarget ddPCR for TcR1 primer
caagtagcgaagcgagcaggac	Genewiz	pTarget ddPCR for TcR2 primer
CTTTCCCTACACGACGCTCTTCCGA TCTagcttccttagctcctgaaaatctcg	Genewiz	NGS AjTn6022-comM upstream primer
CTTTCCCTACACGACGCTCTTCCGA TCTgaaaatgagacgttgatcggcac	Genewiz	NGS AjTn6022-comM downstream primer
GACTGGAGTTCAGACGTGTGCTCT TCCGATCTgccactcttgcttattactgtc	Genewiz	NGS pDonor(AjTn6022)-RE primer
GACTGGAGTTCAGACGTGTGCTCTT CCGATCTttgcaggcatatcttttagtg	Genewiz	NGS pDonor(AjTn6022)-LE primer
gccactcttgcttattactgtc	Genewiz	pDonor(AjTn6022) ddPCR for RE primer
gaaaatgagacgttgatcggcac	Genewiz	pTarget ddPCR Aj-comM downstream primer
CTTTCCCTACACGACGCTCTTCCGA TCTctgggttgaaggctctcaagggc	Genewiz	NGS ZooTsy-comM upstream primer
CTTTCCCTACACGACGCTCTTCCGA TCTgtgtgcttctcaaatgcctgaggtttc	Genewiz	NGS ZooTsy-comM downstream primer
GACTGGAGTTCAGACGTGTGCTCTT CCGATCTctgcaagtaatgcgacattgg	Genewiz	NGS ZooTsy-end1 (for upstream-end1 junction) primer
CTTTCCCTACACGACGCTCTTCCG ATCTcctttatagtcagtgggttatccg	Genewiz	NGS ZooTsy-end1 (for circularized donor junction) primer
GACTGGAGTTCAGACGTGTGCTCT TCCGATCTttcgagatcatgcatgagctcac	Genewiz	NGS KanR-cargo upstream primer
GACTGGAGTTCAGACGTGTGCTCTT CCGATCTtgaggatccaacatttccaatcactag	Genewiz	NGS KanR-cargo downstream primer
ctgcaagtaatgcgacattgg	Genewiz	pDonor(ZooTsy) ddPCR for end1 primer
atcaaaactggtgaaactcacccag	Genewiz	pTarget ddPCR for CmR1 primer
gtgttcacccttgttacaccgttttc	Genewiz	pTarget ddPCR for CmR2 primer
ggtgatgacggtgaaaacctctgac	Genewiz	ZooTsy_CI-sequencing primer
ggtaaatgctgaatcagtacaaaaacaatg	Genewiz	E.coli comM upstream primer for AjTn6022
gattcaatcggctctcgcaaggc	Genewiz	E.coli comM downstream primer for AjTn6022
agtgctgcgatattaagtctggtaaatg	Genewiz	E.coli comM upstream primer for ZooTsy
ccgagagccggttgagataacg	Genewiz	E.coli comM downstream primer for ZooTsy
atgtcactgtcaattgttcatacccgc	Genewiz	E.coli comM ddPCR forward primer
tttttttcgccggatattcatatccgc	Genewiz	E.coli comM ddPCR reverse primer
gtctcgtgggctcggagatgtgtataagagacag	Genewiz	TTISS 1st PCR common primer
gtggcaacctattgttttcttatcatgac	Genewiz	AjTn6022-TTISS_RE 1st primer
AATGATACGGCGACCACCGAGATCTA CACAAGTAGAGACACTCTTTCCCTACA CGACGCTCTTCCGATCTgacactcttatcta ttgctgtaaatgac	IDT	AjTn6022-TTISS_RE 2nd F1 primer
tcctttatagtcagtgggttatccg	Genewiz	ZooTsy-TTISS_End1 1st primer
AATGATACGGCGACCACCGAGATCTACA CAAGTAGAGACACTCTTTCCCTACACGAC GCTCTTCCGATCTgccttgttcgttgctacattggc	IDT	ZooTsy-TTISS_End1 2nd F1 primer
CAAGCAGAAGACGGCATACGAGATCATG ATCGGTCTCGTGGGCTCGGAGATGTGT	Genewiz	TTISS 2nd PCR common R1 primer
CAAGCAGAAGACGGCATACGAGATAGGAT CTAGTCTCGTGGGCTCGGAGATGTGT	Genewiz	TTISS 2nd PCR common R2 primer
CAAGCAGAAGACGGCATACGAGATGACAG TAAGTCTCGTGGGCTCGGAGATGTGT	Genewiz	TTISS 2nd PCR common R3 primer
CAAGCAGAAGACGGCATACGAGATCCTATG CCGTCTCGTGGGCTCGGAGATGTGT	Genewiz	TTISS 2nd PCR common R4 primer
TGCGTTGATGCAATTTCTATGCGCACCCGT	IDT	pTarget ddPCR TcR probe
TGTCCACACCCATGAGTGGACAACTTATGC	IDT	pDonor(CyCAST) ddPCR for RE probe
TCATGATAAGAAAACAATAGGTTGCCACTC	IDT	pDonor(AjTn6022) ddPCR for RE probe
CAACGAACAAGGCAAAAATTCGGATAACCC	IDT	pDonor(ZooTsy) ddPCR for end1 probe
AGGTTTTCACCGTAACACGCCACATCTTGC	IDT	pTarget ddPCR CmR probe
AGGTCTACCCGGCTTAACGATGGTGGGCT	IDT	E.coli comM probe

**Recombinant DNA**		

pUC19 Vector	New England Biolabs	N3041
pBluescript II SK (+)	Agilent	212205
pCDFDuet™-1 DNA	Millipore sigma	71340
pCOLADuet™-1 DNA	Millipore sigma	71406
pACYCDuet™-1 DNA	Millipore sigma	71147
pSC101-Donor	Addgene	#140630
pXT131_TwinStrep-SUMO-ShTniQ	Addgene	#135527

**Software and algorithms**		

Geneious	https://www.geneious.com/	v2022
Blast+	https://blast.ncbi.nlm.nih.gov/Blast.cgi	v2.9.0
Hmmer	http://hmmer.org/	v3.1
iTOL	https://itol.embl.de/	N/A
MAFFT	https://mafft.cbrc.jp/	v7.505
Muscle	https://drive5.com/muscle5/	v5
PyMOL	https://pymol.org/2/	v1.2
Fasttree	http://www.microbesonline.org/fasttree/	v2.1.10
DALI	http://ekhidna2.biocenter.helsinki.fi/dali/README.v5.html	v5
HHsuite	https://github.com/soedinglab/hh-suite	v3.1
psipred	https://github.com/psipred/psipred	v2.6
HHpred webserver	https://toolkit.tuebingen.mpg.de/tools/hhpred	N/A
Mmseqs2	https://github.com/soedinglab/mmseqs2	v12
trimal	http://trimal.cgenomics.org/trimal	v1.2
Alphafold2 Colabfold	https://github.com/sokrypton/ColabFold	N/A
Alphafold2 Multimer colabfold	https://github.com/sokrypton/ColabFold	N/A
Biopython	https://biopython.org/	v1.78
CLANS	http://ftp.tuebingen.mpg.de/pub/protevo/CLANS/	N/A
InsertionReadsCounter	https://doi.org/10.5281/zenodo.7872374	v3.0.0
QuantaSoft™ Software	Bio-Rad	N/A

**Other**		

Bench Protocol	This paper	STAR Methods

### Resource availability

#### Lead contact

Further information and requests for resources and reagents should be directed to and will be fulfilled by the lead contact, Feng Zhang (zhang@broadinstitute.org).

#### Materials availability

Plasmids generated in this study have been deposited to Addgene (Data S3).

### Method details

#### Identification of Tn*7*-like transposons

HMMs of Tn7 proteins (TnsA (PF08721.12, PF08722.12), TnsB (PF00665.27), TnsC (PF05621.12, PF11426.9, PF13401.7), TnsD PF06527.12, PF15978.6), and TnsE (PF18623.2)) were used to search homologs with hmmsearch software^43^ (using option ga_cut) within predicted protein sequences derived from publicly available microbial contigs in the NCBI Genbank and WGS databases, JGI database (projects with stated permission to use), and the MG-RAST database^44^ (all frozen in November 2020). The full database encompasses 521,828,662 contigs in total (contigs greater than 1.5kb), covering 6,932,321,054,498 bp of genomic DNA. 1,617,895 contigs have detectable rpob genes (detected with hmmsearch from TIRGR02013.1 profile), suggesting the diversity is probably reflected by 1.6 million genomes.^45^^,^^46^ Loci were built by mapping the location of the hits into the contig and aggregating hits when they are no further than 20 kb from each other. Loci were selected if they satisfied the following criteria: (i) at least 2 hit genes homologous to 2 distinct Tn7 components, (ii) 2 of the hit genes are in a putative operon, which is operationally defined as 2 codirectional ORFs separated by less than 50 bp of non-coding sequence, and (iii) the hit genes are less than 3 kb from the contig boundary (to remove likely incomplete transposons). Using these criteria, 80,028 loci were obtained from which we extracted and translated 86,517 TnsC homologs. We clustered these homologs at 80% sequence identity over 75% of the protein length (coverage) using MMSeqs2 (v. 12- 113e3)^47^ and obtained 7,789 TnsC homolog representatives.

#### Construction of the phylogenetic tree of TnsC homologs

The protein sequences of representative TnsC homologs were aligned using a method described previously.^48^ Briefly, the protein sequences were clustered at 50% identity and cluster members were extracted and aligned using MUSCLE (version 5).^49^ An all-versus-all comparison of the multiple sequence alignments (MSAs) was computed using HHsearch.^50^ An unweighted pair group method with arithmetic mean (UPGMA) dendrogram was constructed using the HHSearch similarity scores. The dendrogram was used to guide the iterative pairwise alignment of cluster MSAs using HHalign.^50^^,^^51^ Clusters were discarded if they could not be aligned using this approach, leaving 6,988 sequences in the single final alignment. The alignment was first filtered to remove sites with conserved gaps using trimal version 1.2 with the option gappyout.^52^ Finally, aligned sequences from which Walkers A and B were not aligned correctly (any gap in one of the positions) were discarded. This led to an alignment of 6,384 sequences that are used for this study. The alignment was input into FastTree2^53^ with the Whelan-Goldman models of amino acid evolution and gamma-distributed site rates. The tree was visualized and annotated using the interactive tree of life (itol).^54^

#### Annotation of the genomic neighborhoods of TnsC homologs

Representative TnsC homologs were mapped on the respective genomic contigs and genes within 50 kb were extracted. These genes were translated and annotated for specific genes of interest including Cas effectors (Cascade and RAMP components and single protein effector from Class 2 CRISPR-Cas systems) and Cas6 that were annotated using the profiles extracted from DefenseFinder^55^ and hmmsearch with a threshold set at a score of 25 whereby all hits with a score of 25 or greater were selected. Hits were mapped onto leaves of the TnsC homolog tree and subsequently the TniQ homolog tree and displayed with itol (Figures 2 and S2). As TniQ/TnsD can be extremely divergent and therefore not always detected by hmmsearch, a profile-profile comparison was performed using HHalign software, and the TniQ PF06527 profile was used to annotate distant TniQ homologs. TniQ hits were selected if they have a hhsearch probability >= 80. Given the abundance of TniQ, false positive detection of TnsC (AAA-ATpase among cargo), and sometimes multiple Tn7 transposons co-occurring, it is challenging to know which *tniQ/tnsD* is associated with which *tnsC*. For a given *tnsC*, we defined an association with *tniQ/tnsD(s)* if no other *tnsC* homologs were found in the vicinity. If there was a *tnsC* homolog closer than the given *tnsC*, we associated *tniQ/tnsD* to it only if there was a *tnsB* gene operonized with this closer *tnsC*. In total 5,072 *tniQ/tnsD* were found to be associated with TnsC homolog representatives. The presence of these *tniQ/tnsD* were mapped onto the TnsC homolog tree. When multiple *tniQ/tnsD* were found in the vicinity, the two largest were selected and the second selected *tniQ/tnsD* were mapped on an additional barplot indicating the size of the second protein in aa. *tnsE* were detected in relatives of *E. coli* Tn7 but also weakly in Tn6022-like transposons where it is encoded in the far vicinity of the Tn core components (in contrast to *E. coli* Tn7 relatives where *tnsE* is part of a full operon encompassing all Tn core components and *tnsD*). Manual inspection indicated these TnsE remote homologs are encoded near the left end of the transposon far from the other transposon components. Structural comparison from the models predicted by Alphafold2^18^ (Figure S3) confirmed that these hits are TnsE relatives. These remote TnsE were used as a new seed to annotate additional TnsEs using blastp on all translated genes in the vicinity of *tnsC*. 552 TnsE in total were detected, and their presence was mapped on the TnsC homolog tree and on the TniQ homolog tree and displayed using the itol framework.

#### Protein structure prediction and analysis

All structural models were built using Alphafold2 (AF2) software under the colabfold framework installed locally.^17^^,^^18^^,^^19^ Multiple sequence alignments were constructed using colabfold_search on the colabfold database that includes Uniref and environmental protein sequences. Alignments were input into AF2, and three models were generated with 35 recycles. All models were examined using the PyMOL framework (The PyMOL Molecular Graphic System Version 1.2, Schrodinger, LLC), mapping the predicted local distance difference test (plDDT),^18^^,^^56^ a local measure of prediction confidence, on each residue. Regions of the proteins with plDDT less than 50 were not considered. Protein docking prediction was performed using AF2 with the multimeric model.^17^ Results of protein docking were analyzed by examining the predicted aligned error (PAE) matrix^18^ and visualizing the interaction area on PyMOL. The spatial distributions of specific chemical interactions found in protein-protein interactions^57^^,^^58^ were analyzed using PyMOL to validate models when PAE was weak. Predicted structures with high confidence (typically average pLDDT>70) were considered for downstream analysis. Searches for structural similarity were performed using DALI software version 5^25^ using the PDB50 (non-redundant at 50% of sequence identity) and a custom database made from the EBI Alphafold2 database. This database contains Alphafold2 models of Uniref50^47^^,^^59^ extracted from EBI Alphafold2 database (https://alphafold.ebi.ac.uk/) restricted to models having at least 30aa with a plDDT greater than 50 (hereafter called AF2DB50). Hits with Z-scores greater than 5 were retained, and every hit was manually verified by building a structural alignment in PyMOL editing mode.

#### Phylogenetic analysis of TniQ

5,072 *tniQ/tnsD* detected in the vicinity of the Tn7 *tnsC* homolog representatives were translated and aligned using MUSCLE version 5 using the option super5 to cluster the sequences. Alignments were then created for each cluster and merged into a single alignment. The alignment was first filtered to remove sites with conserved gaps using trimal version 1.2 with the option gappyout. As TniQ/TnsD can harbor very divergent C-terminal regions in terms of both sequence and size, the alignment was restricted to the core region of TniQ. To determine the core (roughly corresponding to the first 300aa), the structure of all dual TniQ-TnsD CAST systems was predicted with AF2 and manually aligned structurally (as described above). Core positions were mapped to the sequence alignment and the downstream regions (C-terminal region) were trimmed out. Finally, to filter out misaligned TniQ cores, aligned sequences from which the first CxxC motif of the zing finger were not well aligned (any gap in one of the positions) were discarded. The 4,916 remaining aligned sequences were used to build a tree using FastTree2 with the Whelan-Goldman models of amino acid evolution and gamma-distributed site rates. The presence of *cas* effector genes and *cas6* as well as *tnsE* in the vicinity was shown similarly as for the TnsC tree. Operonized genes with *tniQ/tnsD* (see TniQ partner candidate analysis) were also displayed (light green ring). To investigate the origin of dual TniQ-TnsD, a visual approach and a statistical approach were used. A connector between dual TniQ-TnsD leaves of the tree was drawn and colored based on a rainbow gradient spread across all leaves from the left to the right of the tree. The connector has a uniform color picked from the color assigned to the left-most leaf. If a connector has a color that matched the color both at the starting leaf (left) and the arriving leaf (right), the proteins are closely related. Conversely, any contrast between the colors indicates the proteins are not closely related. For the statistical approach, the branch distances of all dual TniQ-TnsD were extracted and compared with 1000 random branch distances involving nondual TniQ-TnsD across the tree. Branch distances were calculated using the Phylo package from the Biopython library. Biopython package.^60^ Comparison of these distances was done via a T-Test using SciPy python library version 1.0.^61^

#### Analysis of candidate partners of TniQ

Genes operonized with *tniQ/tnsD* were extracted and translated if they were not related to any Tn7 components (TnsA, TnsB, TnsC, TniQ/TnsD, or TnsE). 782 proteins were clustered at 30% of sequence identities and 30% sequences coverages using MMSeqs2 (v. 12- 113e3).^47^ From each cluster, members were aligned together using mafft-linsi.^62^ From each alignment, secondary structures were predicted psipred version 2.6^63^ to ensure compatibility with hhpred, and HMM profiles were built for each using hhmake.^51^ Each hhpred profile was compared to the Pfam protein domain database (preformatted for hhpred and available at https://wwwuser.gwdg.de/∼compbiol/data/hhsuite/databases/hhsuite_dbs/) using hhsearch. Hits with hhsearch probability >= 90 were considered. Candidates operonized with *tniQ/tnsD* were mapped onto the tree (light green ring). The 6 largest groups of candidates were selected based on the conservation of the operonized genes across several adjacent leaves in the TniQ tree and analyzed further. For each candidate, we performed profile analysis (using hhpred webserver) to assess their potential function and structural docking with TnsC and TniQ/TnsD to test for potential interactions (interaction with TnsC could highlight a novel target selector independent from TniQ/TnsD, whereas interaction with TniQ/TnsD could suggest a partner target selector) using Alphafold multimer.

#### TnsF analysis

TnsF from *Acinetobacter johnsonii* Tn6022 (hereafter AjTn6022 and AjTnsF) was chosen as the representative for computational and experimental analysis. HHpred was used to annotate the domain architecture region of AjTnsF and detect the presence of multiple zinc fingers in the N-terminal region (positions 1-180 has a hit to a LIM Zinc-binding domain-containing protein hhprob=97.23) and similarity with tyrosine recombinase in the C-terminal region (positions 328-493 has a hit to a site-specific recombinase IntI4 hhprob=99.14). A structural model of TnsF was obtained using AF2 and split into 3 domains defined by long linkers connecting globular regions and a long N-terminal region encoding several zinc fingers. Each domain was used as a seed for structural similarity search using DALI software across the PDB50 (as described above). Top hits were inspected manually with PyMOL.

#### Mining of TnsF and phylogenetic analysis of TnsF homologs

To search for TnsF relatives, AjTnsF was used as a seed for a psiblast^64^ search for 3 iterations on the NCBI NR database (in August 2022). 22,419 protein hits were extracted and clustered at 80% of sequence identity and 70% of coverage with MMSeqs2 (v. 12- 113e3). A blastall comparison^65^ was performed, and e-values associated with each comparison were input into CLANS software^66^ to cluster hits according to their e-values and draw a graph network representation. Several clusters (point density connected and close to each other) are also connected to each other. AjTn6022 TnsF was mapped onto the graph to identify the cluster to which it belongs in order to define the Tn6022 TnsF cluster. The Tn6022 TnsF cluster connected to another cluster from which several members were extracted and mapped onto genomic contigs. Genomic comparison between these contigs and Tn6022 contigs reveals a distinct system with partial *comM* surrounding the system and with no apparent Tn7 components but other genes operonized with *tnsF*. Hhpred webserver^51^ was used to annotate these genes, revealing the presence of a gene encoding a tyrosine recombinase (yrec), a gene encoding a helix turn helix domain (hth), and a gene encoding a GIY-YIG nuclease. The system was named transposon using Target Selector based on tyrosine (Y) recombinase (Tsy) based on the components operonized with *tnsF*. Hits belonging to the CLANS Tn6022 TnsF cluster and the adjacent cluster harboring the Tsy TnsF were extracted and aligned using MUSCLE version 5 with the super5 algorithm. The alignment of 1,095 protein sequences was further trimmed using trimal version 1.2 (gappyout option) and was input to FastTree2 with the Whelan-Goldman models of amino acid evolution and gamma-distributed site rates. The tree was visualized and annotated using the interactive tree of life (itol). *tnsF* genes were mapped to genomic contigs, and genes in the vicinity (20 kb) were extracted, translated, and further clustered at 30% of sequence identity retaining 50% of coverage using MMSeqs2 (v. 12- 113e3), and each cluster was converted into HMM profile using hhmake and compared to the profile pfam database using HHsearch. The top populated clusters were Tn7 components (*tnsA*, *tnsB*, *tnsC*, and *tniQ/tnsD*) and candidates operonized with Tsy TnsF (*yrec*, *GIY-YIG*). The presence of *tniQ/tnsD* as a marker of Tn7, and the presence of the *yrec* and the *GIY-YIG* nuclease were mapped on the TnsF tree as distinct rings. Split *comM* genes were extracted and translated from AjTn6022 and one Tsy locus and used as seeds to search for the full protein version of Mg chelatase for Tn6022 and Tsy using blastp. The closest Mg chelatase was selected for each of the two systems and used as a seed to detect *comM* pieces in the nucleotide vicinity of each locus using tblastn. The presence of a *comM* hit is indicated as a ring on the TnsF tree. Inspection of the tree shows Tn6022 TnsF is monophyletic (branch support = 0.976). A TnsF from Tsy was extracted from *Zoogloea sp. LCSB751* (ZooTnsF) and the structure was predicted using AF2. Structural comparison between AjTnsF and ZooTnsF was performed manually using a PyMOL framework. The structural similarity search was done using DALI on AF2DB50 (as described above). Hits with a zscore greater than 8 were inspected manually to search for tandem CB+CAT domain architecture using PyMOL.

#### Determination of transposon ends for Tn*6022* and Tsy

Transposon ends for AjTn6022 were determined using Geneious searching for at least one distinct cluster of short repeats (12 nt with 3 mismatches maximum, repeated at least twice in each end) that surround the transposon components including the *tnsA*, *tnsB*, *tnsC*, *tniQ*, *tnsF*, and *tnsE*. Exact end boundaries were then adjusted manually based on local alignment of the clustered repeats area and search for target site duplications. Transposon ends for ZooTsy were determined by prediction based on previous findings about YRec combined with experimental validation (Figure S5). YRec usually works as a dimer to recognize a region with two DNA motifs (each bound by the CB domain of each monomer) and cleave the middle region surrounding these motifs during recombination.^67^ Based on this, we reasoned that in an excision scenario where the cleavage site for excision is at the edge of the partial *comM* gene, one motif should be located within the *comM* gene while the other motif should be located downstream of *comM* in the end of the transposon which would lead to a cleavage site at the transition area between the *comM* gene and the end region. To test this, we first cloned the ends of ZooTsy – 135-bp end1 (the region upstream of *YRec* extending to the border of the 5′-terminal portion *comM*) and 39-bp end2 (the region from the end of the cargo extending to the border of the 3′-terminal portion of *comM*). We then performed transposition assays, initally testing five different extensions (called homology arms (hom): 100-, 50-, 25-, 12-, 0-bp) for each end with the *comM* sequence upstream end1 (hom1) and downstream end2 (hom2) to determine if *comM* itself encoded a motif for end recognition. Based on these results, we concluded that 12 bp are required for hom1 (the requirement for hom2 was inconclusive). We then further refined this initial construct (end1:135bp, end2:39bp, hom1:12bp and hom2:12bp) to determine the minimal requirements for transposition (Figure S5F).

#### Computational analysis of CAST I-D

Given the rarity of CAST I-D in our frozen database, we performed a blastp from CAST I-D Cas10d on the NCBI NR database to fetch additional loci. Only one candidate, from the *Cyanothece sp. PCC 7425* genome (CyCAST), harbors a predicted active HD and Cas3’. The CRISPR-array of CyCast was predicted using minced^68^ with default parameters, and transposon ends were determined as described above. Exact end boundaries were then adjusted manually based on local alignment of the clustered repeats area and search for target site duplication. CAST I-D Cas10d was used as a seed to search for homologs using blastp on the NCBI NR database. An initial phylogenetic tree (using FastTree) was done from the sequence alignment (using mafft-linsi) of the hits, and the subtree harboring all CAST I-D Cas10d and a few non-CAST I-D Cas10d were extracted. A structural model of CyCAST Cas10d was obtained using AF2 and used to determine the position of the catalytic sites of the HD nuclease domain. These positions were mapped onto the alignment from which a sub-alignment encompassing these positions and their local sequential vicinity was built and juxtaposed next to the tree.

#### Plasmid construction

All plasmids used in this study are described in Data S3. For type I-D CyCAST experiments, genes encoding *Cyanothece sp. PCC 7425* (ASM2204v1_genomic|CP001344.1|5374574|) TnsAB-TnsC-TniQ-TnsD, and Cas10d-Csc2-Cas5-Cas6 were synthesized (Twist Bioscience) and cloned into pCDFDuet-1 with the appropriate spacer flanked by two CRISPR direct repeats, yielding pHelper(CyCAST). Gene fragments encoding both transposon ends (Genewiz) were cloned into pBluescript II SK (+) (Agilent), yielding pDonor(CyCAST). For PAM screens, a 0.5-kb exon fragment amplified from human *EMX1* was inserted between the transposon ends as a mock non-functional cargo in *E. coli*. For AjTn6022 experiments, genes encoding *Acinetobacter johnsonii* Tn6022 locus TnsA-TnsB-TnsC-TniQ-TnsF were synthesized (Twist Bioscience) and cloned into pCDFDuet-1. Predicted AjTn6022 transposon ends (RE and LE) were synthesized (Genewiz) and cloned into pBluescript II SK (+) with a kanamycin resistance gene to obtain pDonor(AjTn6022). For ZooTsy experiments, genes encoding *Zoogloea sp. LCSB751* Tsy locus YRec-HTH-TnsF-GIY-YIG nuclease were synthesized (Twist Bioscience) and cloned into pCDFDuet-1. Predicted ZooTsy transposon ends (end1 and end2) were synthesized (Genewiz) and cloned to obtain pDonor(ZooTsy). Throughout the study, pBluescript-donor was used for CyCAST, and pR6K-donor (backbone: Addgene#127924) was used for AjTn6022 and ZooTsy experiments, except for Tagmentation-based Tag Integration Site Sequencing (TTISS) analyses, for which pSC101-donor was used (backbone: Addgene#140630). To construct pHelper variants for CyCAST, AjTn6022, and ZooTsy, the Q5 Site-Directed Mutagenesis Kit (NEB) was used. For pHelper(AjTn6022) TniQ point mutants, a ribosome binding site and a partial TnsF N-terminal coding sequence was inserted to separate overlapped ORFs of TniQ and TnsF, and mutations were introduced into TniQ. The tRNA-Val gene of pTarget(PmcCAST) (Addgene#168163) was replaced by either tRNA-Leu gene of *Cyanothece*, *comM* gene fragment of *A. johnsonii*, or *comM* gene fragment of *Zoogloea sp*. *LCSB751*, yielding pTarget(CyCAST), pTarget(AjTn6022), or pTarget(ZooTsy), respectively. For TniQ and TnsF protein purification, TniQ of AvCAST, PmcCAST, CyCAST, Tn7017, and AjTn6022 were individually cloned into ColE1-based pTwinStrep-SUMO bacterial expression vector, and ShCAST (Addgene#135527) were used. TnsF of AjTn6022 and nuclease-dead TnsF-Y584F mutant of ZooTsy were individually cloned into ColE1-based pTwinStrep-bdSUMO bacterial expression vector.

#### *E. coli* transposition assays

For CyCAST, 100 ng of pHelper(CyCAST) was co-electroporated with 100 ng of pDonor(CyCAST) and 100 ng of pTarget(CyCAST) into BL21(DE3) electrocompetent cells (Sigma) and plated on 100 μg/ml carbenicillin, 50 μg/ml spectinomycin, and 50 μg/ml chloramphenicol containing LB-agar plates. After incubation for 17 hours at 37°C, all colonies were scraped from the plates, and a portion was re-plated on 0.1 mM IPTG supplemented triple antibiotic LB-agar plates to induce protein expression. Cells were incubated for an additional 17 hours at 37°C. All colonies were scraped, and the plasmid DNA was purified using QIAprep Spin Miniprep Kit (QIAGEN). Insertions were identified by PCR using Q5 High-Fidelity DNA polymerase (NEB). Insertion products containing the 6N sequence were amplified and sequenced using a MiSeq Reagent Kit v2, 300-cycle (Illumina). PAM and insert position were characterized as previously described.^8^ The frequency of insertions was determined with droplet digital PCR (ddPCR) as described below with 1 pg to 10 ng template plasmid DNA for 20 μL ddPCR reaction. AjTn6022 and ZooTsy were analyzed in the same manner, but a constitutive lac promoter was used for expression of proteins. In brief, co-electroporated transformants were incubated with triple antibiotic (50 μg/ml kanamycin, 50 μg/ml spectinomycin, and 50 μg/ml chloramphenicol) containing LB-agar plates for 17 hours at 37°C, and all colonies were scraped for downstream analysis. Experiments were performed with three biological replicates. All data points are shown with an error bar showing standard deviation, and statistical significance was assessed by t-test.

#### Nanopore long-read sequencing to determine plasmid structure

To isolate pInsert and determine its structure, the pR6K-donor was utilized. For AjTn6022, pHelper(AjTn6022), pR6K-donor(AjTn6022), and pTarget(AjTn6022) were co-electroporated into Pir1+ *E. coli*. Cells were recovered for 1 hour and plated on 50 μg/ml kanamycin, 50 μg/ml spectinomycin, and 50 μg/ml chloramphenicol containing LB-agar plates. Cells were harvested 5 days after incubation at room temperature and subjected to mini-prep by QIAprep Spin Miniprep Kit (QIAGEN). Note that we avoided incubation at 37°C to prevent transposed products from being actively resolved to enable detection of replicative transposition. To recover transposed pInsert products, 100 ng of mini-prep product was electroporated into Endura Competent Cells (Lucigen). Cells were recovered for 1 hour and plated on 50 μg/ml kanamycin and 50 μg/ml chloramphenicol containing LB-agar plates, and further incubated at room temperature for 5 days. Donor insertion on pTarget was confirmed by Sanger sequencing of mini-prep products for 4 colonies. In parallel, all colonies were harvested and subjected to mini-prep, followed by amplification-free nanopore sequencing library preparation (Oxford Nanopore Technologies SQK-LSK109). Briefly, mini-prep products were linearized by BstZ17I, followed by end-prep and subsequent ligation of sequencing adapters. Resulting libraries were loaded on a MinION R9 flow cell and sequenced. Sequence reads containing 25 bp of *comM* gene fragment, 25 bp of LE, and 25 bp of RE (up to 2 bp mismatches were allowed for each component) were filtered for further analysis, thereby discarding low-quality and contaminating gDNA reads. ZooTsy was analyzed in the same manner with cognate plasmids. For quality control, reads over 2.5 kb with QScore >7 were filtered. To determine the structure of pInsert, the reads were mapped on the expected simple insertion product (Figure S5D). To count simple insertions, nanopore sequencing reads were analyzed using the following pipeline (Figures S5C and S5E): the reads which contain both (1) the downstream edge 30-bp sequence of cloned *comM* 100-bp fragment (AGCGGGCCGGGAACTCGGCGCGGCGGGCTG) and (2) 30-bp end2 sequence (GGACTGGGATTCTCCAATATTCCTTAGCGC) were further extracted by the insertion reads counter. Among those reads, the ones which have (3) the upstream edge 30-bp sequence of the cloned *comM* fragment (CACGGCTTCGACCGCAGCACTGGTCGGTGG) and (4) the 30-bp sequence of the cargo (GTCGGGGGGATCCACTAGTGAGCTCATGCA) were extracted by the counter and used for mapping on the expected simple insertion. Based on the distance between the upstream edge of *comM* and cargo, each selected read was counted as a simple insertion.

#### Tsy circular intermediate isolation assays

100 ng of pHelper(ZooTsy) was co-electroporated with 100 ng of lacZɑ backbone pDonor(ZooTsy) and 100 ng of pACYC backbone vector into BL21(DE3) electrocompetent cells (Sigma) and plated on 50 μg/ml kanamycin, 50 μg/ml spectinomycin, and 50 μg/ml chloramphenicol containing LB-agar plates. After incubation for 17 hours at 37°C, all colonies were scraped from the plates, and the plasmid DNA was purified using QIAprep Spin Miniprep Kit (QIAGEN). 100 pg of mini-prep product was used for re-transformation of NEB 10-beta Competent *E. coli* (NEB), and the cells were plated on S-Gal (Sigma) and 50 μg/ml kanamycin containing LB-agar plates for blue/white selection. After incubation for 17 hours at 37°C, white colonies were picked up and further cultured for 17 hours at 37°C, and the plasmid DNA was purified using QIAprep Spin Miniprep Kit (QIAGEN). The original lacZɑ backbone pDonor(ZooTsy) and the circularized intermediate (CI) were digested with NruI (NEB) for an hour at 37°C and loaded on E-Gel Ex Agarose Gels 1%. The junction of end1 and end2 was confirmed by Sanger sequencing of mini-prep products for 4 white colonies. In parallel, isolated products from 24 white colonies were combined and subjected to amplification-free nanopore sequencing library preparation (Oxford Nanopore Technologies SQK-LSK109). Briefly, mini-prep products were linearized by NruI, followed by end-prep and subsequent ligation of sequencing adapters. Resulting libraries were loaded on a MinION R9 flow cell and sequenced.

#### TTISS for insertion specificity analysis

Endura Competent Cells were transformed for transposition assay as mentioned above, with the following modifications: 100 ng of a temperature-sensitive pSC101-donor was used. After incubation on triple antibiotic LB-agar plates, cells were re-plated and grown for 12 hours at 43°C to prevent unintended amplification of donor plasmids in the following Tagmentation-based Tag Integration Site Sequencing (TTISS) analysis.^36^ Genomic DNA was extracted from hundreds of colonies on a LB-agar petri dish, and 500 ng genomic DNA (from approximately 10^8^ *E. coli* cells) was tagmented with Tn5, and re-purified by Wizard SV Gel and PCR Clean-Up System (Promega). Tagmented DNA samples were amplified using two rounds (in total 37 cycles) of PCR with KOD Hot Start DNA Polymerase (Millipore) using a Tn5 adapter-specific primer and nested primers within the DNA donor (Data S3). PCR products from 4 different experimental conditions were pooled together, purified, and the resulting libraries were sequenced using a NextSeq v2 kit (Illumina), 75 cycle kit with 45 forward cycles and 30 reverse cycles. Read pairs with R1 containing the terminal 29 bp of the AjTn6022 transposon RE sequence were filtered for further analysis and trimmed of the transposon sequence for alignment to the *E. coli* genome (CP011113.2) and pTarget(AjTn6022). Filtered and trimmed reads were aligned using the established BWA aligner pipeline.^69^ The resulting SAM files were exported for further analysis. Aligned R1 reads with length of 16 bp (remaining R1 length after trimming of 29 bp of transposon RE sequence) and SAM flags 99 and 147 (for mapped reads within the insert size and in correct orientation) were used to determine the correct transposon insertion reads. Reads with a single insertion position at the *AjcomM* on pTarget and *E. coli* endogenous *comM* insertion sites were considered on-target, while remaining reads were counted as off-targets. For ZooTsy, the resulting libraries were sequenced using a MiSeq v2 kit with 75 forward cycles and 75 reverse cycles. Read pairs with R1 containing the terminal 36 bp of the ZooTsy end1 sequence were filtered for further analysis and trimmed of the transposon sequence for alignment to the *E. coli* genome and pTarget(ZooTsy). Filtered and trimmed reads were aligned, and the resulting SAM files were exported for further analysis. Aligned R1 reads with length of 39 bp (remaining R1 length after trimming of 36 bp of transposon end1 sequence) and SAM flags 99 and 147 were used to determine the correct transposon insertion reads.

#### Droplet digital PCR reactions

Insertion events were quantified using insertion specific primers and a donor specific probe (Data S3). ddPCR Supermix for Probes (No dUTP) (BioRad), primers (900 nM each), a probe (250 nM), and template DNA were combined into 20 mL reactions, and droplets were generated with 70 μL of Droplet Generation Oil for Probes (BioRad) using the QX200 Droplet Generator (BioRad). Thermal cycling conditions for ddPCR reactions were as follows: 1 cycle, 95°C, 10 min; 40 cycles, 94°C, 30 s, 58°C, 1 min; 1 cycle, 98°C, 10 mins; 4°C hold. PCR products were read with a QX200 Droplet Reader, and the absolute concentrations of inserts were determined using QuantaSoft (v1.6.6.0320). Total template (genome or target plasmid) amount was also quantified through this process, and insertion frequency was calculated as inserts/template.

#### Purification of TniQ and TnsF proteins

Each protein expression vector was transformed into BL21(DE3) Competent Cells (NEB). 4 mL of starter culture was grown in TB supplemented with 100 μg/ml ampicillin for 12 h, which was used to inoculate 2 L of TB for growth at 37°C and 150 rpm until an OD600 of 0.6. Protein expression was induced by supplementation with IPTG to a final concentration of 0.5 mM. The cells were incubated at 16°C for 16 h for protein expression, and then harvested by centrifugation for 20 min at 4°C at 4000 rpm (Beckman Coulter Avanti J-E, rotor JLA9.100). All subsequent steps were performed at 4°C. Cell pellet was resuspended in 200 mL of lysis buffer (50 mM Tris-HCl, 500 mM NaCl, 5% glycerol, 1 mM DTT, pH 8.0) supplemented with cOmplete Protease Inhibitor Cocktail (Millipore sigma 4693116001). Cells were disrupted by the LM20 Microfluidizer system at 28,000 PSI. Lysate was cleared by centrifugation for 30 min at 4°C at 9000 rpm (Beckman Coulter Avanti J-E, rotor JLA-10.500). The cleared lysate was applied to 1 mL of packed Strep-Tactin Sepharose resin (IBA) and incubated with rotation for 1 h, followed by washing of the protein-bound beads in 50 mL of lysis buffer. The proteins were cleaved off by Ulp1 SUMO protease OR SENP at 4°C for 16 h. Resulting proteins were concentrated by an Amicon Ultra Centrifugal Filter Units (Millipore), and protein concentration was estimated by NuPAGE (Invitrogen) and eStain L1 Protein Staining System (GenScript). The concentrated protein was loaded onto a gel filtration column (Superdex 200 Increase 10/300 GL, GE Healthcare) equilibrated with storage buffer (50 mM Tris-HCl, 250 mM NaCl, 5% glycerol, 1 mM DTT, pH 8.0) via FPLC. The resulting fractions from gel filtration were analyzed and the fractions containing the protein were pooled and stored at -80°C. To purify TwinStrep-bdSUMO-TnsF from AjTn6022, the bound bdSUMO-tagged proteins were eluted in 10 mL of lysis buffer supplemented with 2.5 mM desthiobiotin (Sigma) instead of digestion by SENP.

#### Pull-down experiments for detecting AjTn6022 TniQ-TnsF interactions

TwinStrep-bdSUMO-TnsF was mixed with each purified TniQ protein in the assembly buffer (25 mM Tris-HCl, 250 mM NaCl, 1 mM DTT, pH 8.0) and incubated at 37°C for 1 hour. The reaction was mixed with pre-washed Strep-Tactin Magnetic Microbeads (IBA) and further incubated at 4°C for 30 min. The beads were washed three times with the wash buffer (25 mM Tris-HCl, 250 mM NaCl, 1 mM DTT, 0.05% Tween20, pH 8.0). Then, the protein complexes on the beads were eluted and denatured in NuPAGE LDS sample buffer at 95°C for 10 min. Samples were loaded on NuPAGE 4 to 12%, Bis-Tris Gel, separated by electrophoresis, and stained with Imperial Protein Stain (ThermoFisher Scientific).

#### Electrophoretic mobility shift assay for detecting TnsF-DNA interactions

Purified PCR amplicons and oligos (HPLC purified, IDT) were used as DNA probes for EMSA assays. For DNA-protein complex assembly, 20 ng DNA probe was mixed with purified AjTn6022 TnsF protein or ZooTsy nuclease-dead TnsF-Y584F mutant protein at different DNA:protein molecular ratio in assembly buffer (25 mM Tris-HCl, 50 mM NaCl, 1% glycerol, 5 mM MgCl_2_, pH 8.0), and incubated at 37°C for 1 hour. Samples were mixed with Novex Hi-Density TBE Sample Buffer (ThermoFischer Scientific) and loaded on pre-equilibrated Novex 6% TBE Gel (ThermoFischer Scientific) in 0.5X TBE buffer for electrophoresis. Gel images were captured by SYBR Gold-staining.

### Quantification and statistical analysis

#### Quantification of ddPCR data

Frequency of transposition event was determined by ddPCR using QuantaSoft (v1.6.6.0320). Copy number of generated transposon and target junction was measured by transposon end specific FAM probes (Data S3). Copy number of the target site (on genome or target plasmid) was also quantified through this process using specific FAM probes at independent ddPCR reactions and insertion frequency was calculated as inserts/template ^∗^ 100 and displayed in %. All data points are shown with an error bar showing standard deviation, and statistical significance was assessed by two-tailed t-test.

## Data Availability

• All Illumina NGS and Oxford Nanopore Technologies (ONT) sequencing data generated from this publication have been deposited and are publicly available as of the date of publication. Accession numbers are listed in the key resources table. • All original code for transposition junction NGS reads analysis has been deposited to GitHub and Zenodo. DOI are listed in the key resources table. • Any additional information required to reanalyze the data reported in this paper is available from the lead contact upon request.

## Supplemental information

Document S1. Figures S1–S7

Data S1. An archive containing data used in this study related to Figures 2 and S2–S4 Data includes: (A) LOCIannot.txt: Tn7-like loci used. (B) Tn7_clade.lst: nodes belonging to the Tn7 clade. (C) TNSC.csv: information about TNSC representatives. (D) TNSC.tree: tree of TNSC representatives in newick format. (E) TNSC_for_tree.fasta.alig.trim.walkers: TnsC alignment used to build the tree. (F) TNIQ.csv: information about TniQ representatives. (G) TNIQ.tree: tree of TniQ in newick format. (H) TNIQ_for_tree.fasta.alig.trim.core: TniQ alignment used to build the tree. (I) TNSF_vin.csv: information about TnsF and relative systems. (J) TNSF.tree: tree of TnsF in newick format. (K) itol.link: links to interactive trees displaying features described in the paper are available via Itol TNSC: https://itol.embl.de/tree/13783123246421658282398 TNIQ: https://itol.embl.de/tree/13783123282721658801217 TNSF: https://itol.embl.de/tree/13783123254041660094518

Data S2. Raw gel images, related to Figure 5C (A) Gel showing purified TniQ proteins. (B) Uncropped gel shown in Figure 5C.

Data S3. List of reagents used in this study, related to STAR Methods Reagents include plasmids (A), primers (B), and probes (C), sequences of transposon ends are also included (D).

Document S2. Article plus supplemental information
