# Supplementary material for: Modularity and diversity of target selectors in Tn7 transposons
Source: Mol Cell. 2023 Jun 15;83(12):2122–2136.e10. doi: 10.1016/j.molcel.2023.05.013 (PMC10293859; doi:10.1016/j.molcel.2023.05.013)
Supplement: Document S1. Figures S1–S7 [file mmc1.pdf]

Molecular Cell, Volume *83*

Supplemental information

Modularity and diversity of target selectors in Tn7 transposons

Guilhem Faure, Makoto Saito, Sean Benler, Iris Peng, Yuri I. Wolf, Jonathan Strecker, Han Altae-Tran, Edwin Neumann, David Li, Kira S. Makarova, Rhiannon K. Macrae, Eugene V. Koonin, and Feng Zhang

Supplementary Notes

Supplementary Figure Legends

Figure S1. Identification and characterization of CAST I-D, related to Figure 3
(A) Locus architectures PmcCAST I-B2 and CAST I-D. Gray rectangles show predicted ends, light gray genes indicate predicted homing sites. Cargo areas are summarized by a dark gray rectangle. Cascade components are shown in purple; adaptation module and *cas3d* are shown in lighter purple. CRISPR arrays are shown by a dark gray vertical rectangle for repeats and dark purple diamonds for spacers. Contigs and coordinates are indicated for CAST I-D loci (the absence of one of locus ends and coordinates indicates the edge of the contig). NCBI accessions of Cas10d proteins are written below each *cas10d* gene. **(B)** Cas10d subtree restricted to CAST I-D and close relatives. Local alignment of the HD nuclease catalytic sites is shown on the right of the tree and the presence or absence of Tn7 components is indicated. Right, structural model of CyCAST Cas10d colored by domain architecture. The inset shows the detailed area of the catalytic pocket. **(C)** Weblogo representation of protospacer adjacent motif (PAM) of CyCAST. PAM was determined by targeting a pTarget library containing protospacer adjacent motif 1 (PSP1) flanked by a 6N motif upstream of the protospacer with a single CyCAST protein expression vector (pHelper). **(D)** CyCAST RNA-guided insertion positions and directionality identified by deep sequencing with four different primer pairs. **(E)** Docking predictions of CyCAST TnsC (coral) with TniQ (green) and TnsD (pink). Insets are zoom ins of two regions of interaction between TnsC with TniQ and TnsD. The C-terminal portion of the TniQ core region is disordered and truncated compared to the corresponding regions of CAST I-D TnsD and its relatives in CAST I-B2, suggesting that the interaction between TniQ and Cascade I-D is unstable and that TniQ serves as a facilitator rather than an essential scaffold for the interaction between Cascade I-D and TnsC. **(F)** Protein-mediated insertion by CyCAST in the absence of TniQ at tRNA-leu gene on target plasmid, as identified by deep sequencing.

Figure S2. Small TniQ are associated with additional target selectors, related to Figure 4.
(A) Structural comparison of Tn7 TnsD and CAST TniQ/TnsD proteins. TniQ are involved in RNA-guided transposition and partner with the with the Cas effector for RNA-guided transposition, whereas TnsD are involved in protein-guided transposition. Hel1, ZF, and linker helix are conserved in all TniQ/TnsD and are colored using the same palette as in **Figure 4.** Hel2 (shown between the two dashed lines) has two distinct folds, one colored in rainbow by secondary structure, has structural similarity to XRE TF and is shared between Tn7 TnsD and CAST TniQ/TnsD from I-B and I-D (top row), the other colored in red is found in CAST I-F TniQ/TnsD (bottom row). The C-terminal regions of TniQ/TnsD are partially truncated for visualization in TniQ/TnsD harboring long extensions that are partially shown in pink. **(B)** Phylogenetic tree of the core region of TniQ/TnsD (see **Methods**). Rings around the tree show the presence of a particular gene or a feature in the neighborhood of *tniQ/tnsD* within the genomic contig. From

inner to outer ring: TniQ/TnsD protein size is shown as a bar proportional to the length of TniQ/TnsD, presence of *tnsE* is shown in red, *cas* effectors and *cas6* genes are shown in purple, the presence of a gene operonized with *tniQ/tnsD* is shown in light green. Various known transposons are annotated around the tree including known CAST systems. Red boxes highlight areas of interest, and gene architectures of these systems are shown in **D**. Dual *tniQ-tnsD* are highlighted via a colored connector, where closely related proteins originate and arrive within the same-colored region (see Methods) **(C)** Left. Protein size distribution of TniQ/TnsD in single TniQ/TnsD systems (2905 loci; in red) and dual TniQ-TnsD systems (1029 loci; in green). From the 4 peaks, we defined 4 groups (delineated by dashed lines): Group 1 encompasses proteins that are smaller than 220aa and are mainly seen in CAST V-K systems. The second group of proteins range from 220aa to 400aa and encode the TniQ core region with no or only a short C-terminal addition, which suggests they probably cannot recognize target sites alone and need a partner to perform target selection. The proteins from group 3, which are the most widespread, range from 400aa to 550aa and encode a C-terminal extension that is likely long enough to recognize target sites. Finally, group 4 encompasses proteins larger than 550aa and which harbor very large C-terminal regions, suggesting they bind longer target sites and/or encode additional functions. Right. Protein size comparison of TniQ/TnsD in dual TniQ-TnsD systems. Y axis indicates the protein size of the smaller of the TniQ/TnsD tandem; X axis indicates the size of the larger one. Solid lines segregate the different groups by protein size. Number of occurrences is indicated for each of the groups. **(D)** Schematics of locus architecture for systems of interest (corresponding to systems boxed in red in **B**). One example of a system harboring divergent dual TniQ-TnsD in which the two proteins share less than 20% of sequence identity. Five examples of systems with conserved candidates (in green) operonized with a *tniQ/tnsD*. Below the first candidate is a docking prediction between the candidate (light green), TnsC (coral) and TniQ (dark green). TniQ is truncated for visualization purposes.

Figure S3. TnsE likely targets replication forks of conjugative plasmids, related to Figure 4. **(A)** Phylogenetic tree of TnsC homologs and presence of TnsE (in red). The two distinct TnsE-containing branches are represented by Tn7 *E. coli* (Tn7) and Tn6022. These two groups of transposons harbor distinct *tnsE*s distantly related and only detectable by profile profile comparison (hhpred probability >=90) (see **Methods**), suggesting an ancient origin of *tnsE* in the Tn7 family. **(B)** Locus architecture of Tn7 (top) and Tn6022 (bottom) encoding *tnsE*. Below each locus, the predicted domain architecture of their respective TnsE proteins is shown. Both TnsEs have two single strand DNA binding like domains (SSBa and SSBb) in the N-terminal region and a double strand DNA binding domain (DBD) in the C-terminal region that has been solved experimentally (PDB: 5D17). The SSBs are deduced from structural modeling and structural mining (**C** and **D**). **(C)** Structural modeling of TnsE Tn7 and Tn6022 indicates similar domain architecture despite their low sequence similarity (8% sequence identity). **(D)** Structural superimposition of TnsE N-terminal domains (pink and orange) with PriB (light and dark blue) for

Tn7 TnsE (left) and Tn6022 TnsE (right), suggesting TnsE contains 2 SSB domains. The second domain is split into three regions separated by a linker (yellow) and a domain of unknown function.

Figure S4. Characterization of TnsF, related to Figures 5 and 6.
(A) Rationale for TnsF nomenclature, as part of the progression following the known proteins TnsA, TnsB, TnsC, TnsD, and TnsE. We note however that TnsF has been used once previously to describe a protein in Tn6230[69] (top); however, profile domain analysis (hhpred probabilities >90) showed that this protein is a TnsD, rather than a previously unnamed protein. We therefore named the target selector we found TnsF. We note however, that TnsF has been reported in the literature in a Tn6022[29], although it was annotated as orf3, reflecting its lack of characterization. **(B)** Comparison of predicted structures of the CB1, CB2, and pCAT domains of TnsF with the homodimer tyrosine recombinase (Yrec) XerH structure bound to DNA. Each Yrec monomer contains one CB and one CAT (monomers are circled in dashed red lines). The CB and CAT domains of the Yrec monomer bind the attachment site and dimerize with the corresponding domains of another Yrec monomer. Structural similarity between CB and CAT of Yrec and CB and pCAT of TnsF is shown via matching secondary structural colors. Gray colored regions are found only in Yrec and not in TnsF. **(C)** Nanopore long-read sequencing to characterize the structure of pInsert from *Acinetobacter johnsonii* Tn6022 (AjTn6022). Left: Nanopore sequencing reads mapped to the reference sequence of an AjTn6022 simple insertion on pTarget. Right: Nanopore sequencing reads mapped to the reference sequence of an AjTn6022 cointegrate on pTarget. See also Figure 5B. **(D)** Sanger sequencing chromatograms for RE and LE junctions of a simple insertion at AjTn6022 attachment site in *comM* 100 bp-fragment on pTarget. "CCCGC" is a target site duplication (TSD). See also Figure 5B. **(E)** Predicted domain architectures and structures of AjTnsF (Tn6022) (top) and ZooTnsF (Tsy) (bottom). ZooTnsF contains a complete catalytic domain (CAT in purple and orange). The position of the catalytic tyrosine residue (Y535) is annotated in ZooTnsF and in the structure. **(F)** Phylogenetic tree built from TnsF homologs extracted from the CLANS analysis (see **Methods**). Rings around the tree show the presence of a particular gene or a feature in the neighborhood of *tnsF* within the genomic contig. From inner to outer ring: presence of *comM* fragments in the vicinity of *tnsF* in dark purple, presence of a tyrosine recombinase gene (*yrec*) in light purple, presence of *tniQ* in green, and presence of gene encoding a GIY-YIG nuclease in light blue. TnsF protein size is shown in pink as a bar proportional to its length.

Figure S5. Characterization of ZooTsy transposition, related to Figure 6.
(A) ZooTsy transposition assay. Left top: Schematic of the ZooTsy donor and target *comM* fragment for transposition assay in *E. coli*. Left bottom: Representative PCR amplicon gel images for upstream-end1, downstream-end2, and circularized intermediate (CI) junction products. pHelper contains all Tsy components (YRec, HTH, TnsF, and GIY-YIG). Right top row: Schematic of the Tsy CI isolation assay with a lacZα backbone pDonor. *E. coli* was transformed with pHelper and lacZα donor. 24 hours later, plasmids were prepped and used for re-

transformation. The resulting transformants were plated on blue/white selection plates. Right middle row: left, Representative images of *E. coli* colonies on blue/white selection plates; middle, representative gel image showing the original lacZα donor and isolated CI after linearization by NruI restriction enzyme; right, structure of the CI as characterized by nanopore long-read sequencing. Right bottom row: Sanger sequencing chromatograms for end1 and end2 junctions of the CI. **(B)** Schematic of transposition assay with R6K origin pDonor to isolate pInsert for nanopore long-read sequencing. **(C)** Schematic of nanopore sequencing reads analysis pipeline. **(D)** Structure of pInsert as characterized by nanopore long-read sequencing. **(E)** Top: Determinants of donor end1 for ZooTsy transposition. Representative gel images of PCR amplicons from the upstream-end1 (u-end1), downstream-end2 (d-end2), and CI junctions. Six donor constructs with different end1 lengths (hom1:12 bp, end1:135-85 bp, end2: 39 bp, hom2: 12 bp) were tested. Bottom: Determinants of donor end2 for ZooTsy transposition. Representative gel images of PCR amplicons from the u-end1, d-end2, and CI junctions. Seven donor constructs with different end2 lengths (hom1:12 bp, end1:135 bp, end2: 39-0 bp, hom2: 12 bp) were tested. **(F)** Donor sequence determinants for ZooTsy transposition. Top: Schematic of ZooTsy donor. Bottom: Representative gel images showing PCR amplicons from the u-end1, d-end2, and CI junctions. Sixteen donor constructs with systematic combinations of four elements (hom1, end1, end2 and hom2) were tested. **(G)** Representative gel images showing PCR amplicons from the u-end1, d-end2, and CI junctions (see Figure 6B). **(H)** Requirements of tyrosine recombinase activities of YRec and TnsF for ZooTsy transposition activity. Quantification of u-end1 junction formation by ddPCR. Experiments were performed with three biological replicates. All data points are shown with an error bar showing standard deviation, and statistical significance was assessed by t-test; **** $p<0.0001$.

Figure S6. Analysis of AjTn6022 and ZooTsy insertions, related to Figures 5 and 6.
(A and B) Tagmentation-based tag integration site sequencing (TTISS) analysis in the absence of TnsF and/or presence of the pTarget for AjTn6022 **(A)** and ZooTsy **(B)**. Filtered reads are mapped on CP011113.2 (Strain RR1, HB101 RecA+), complete genome for HB101-derived Endura competent cells and pTarget(AjTn6022) and pTarget(ZooTsy) sequence. **(C)** Insertion frequency of AjTn6022 into each *comM* gene fragment on pTarget. Twelve different *comM* fragments were tested. Ux indicates the number (x) of upstream bp in the fragment; Dy indicates the number (y) of downstream bp in the fragment. U0D0 has no *comM* fragment. **(D)** Insertion frequency of ZooTsy into each *comM* gene fragment on pTarget. Twenty-two different *comM* fragments were tested. Ux indicates the number (x) of upstream bp in the fragment; Dy indicates the number (y) of downstream bp in the fragment. U0D0 has no *comM* fragment. Experiments were performed with three biological replicates. All data points are shown with an error bar showing standard deviation, and statistical significance was assessed by t-test. ***, $p<0.001$; ****, $p<0.0001$; n.s., not significant.

Figure S7. Analysis of the TnsF binding motif, related to Figure 6.

(A) Electrophoretic mobility shift assay to assess purified AjTnsF binding to a 200-bp *AjcomM* fragment containing either the wildtype WT or mutated (various 10-bp mutation constructs) predicted TnsF binding motif region. **(B)** Electrophoretic mobility shift assay to assess purified ZooTnsF_Y584F binding to a 200-bp *ZoocomM* fragment containing either the WT or mutated (various 10-bp mutation constructs) predicted TnsF binding motif region. **(C)** Insertion frequency of AjTn6022 into pTarget with a 200-bp fragment of *AjcomM* harboring the indicated 10-bp-mutation in the TnsF binding motif. **(D)** Insertion frequency of ZooTsy into pTarget with a 200-bp fragment of Zoo *comM* harboring the indicated 10-bp mutations in the TnsF binding motif. Experiments were performed with three biological replicates. All data points are shown with an error bar showing standard deviation, and statistical significance was assessed by t-test. ****, $p<0.0001$.

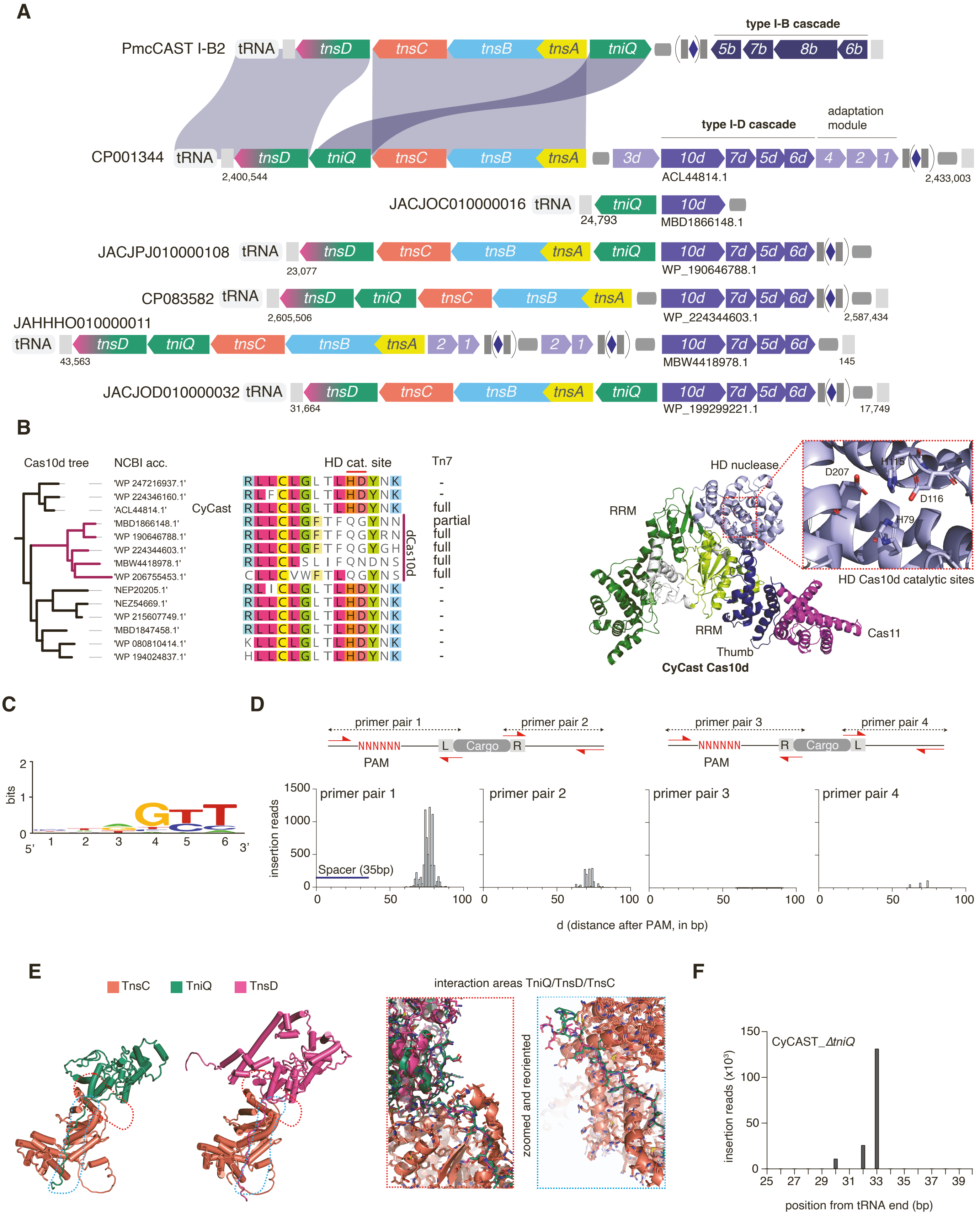

A
PmcCAST I-B2
tRNA
tnsD
tnsC
tnsB
tnsA
tniQ
type I-B cascade
5b
7b
8b
6b
CP001344
type I-D cascade
adaptation module
3d
10d
7d
5d
6d
4
2
1
2,400,544
ACL44814.1
2,433,003
JACJOC010000016
24,793
MBD1866148.1
JACJPJ010000108
23,077
WP_190646788.1
CP083582
2,605,506
WP_224344603.1
2,587,434
JAHHHO010000011
43,563
MBW4418978.1
145
JACJOD010000032
31,664
WP_199299221.1
17,749
B
Cas10d tree
NCBI acc.
HD cat. site
Tn7
CyCast
dCas10d
full
partial
HD nuclease
RRM
Thumb
Cas11
CyCast Cas10d
HD Cas10d catalytic sites
D207
H115
D116
H79
C
bits
D
primer pair 1
primer pair 2
primer pair 3
primer pair 4
NNNNNN
PAM
Cargo
L
R
insertion reads
Spacer (35bp)
d (distance after PAM, in bp)
E
TnsC
TniQ
TnsD
interaction areas TniQ/TnsD/TnsC
zoomed and reoriented
F
CyCAST_ΔtniQ
insertion reads (x10³)
position from tRNA end (bp)

Figure S1

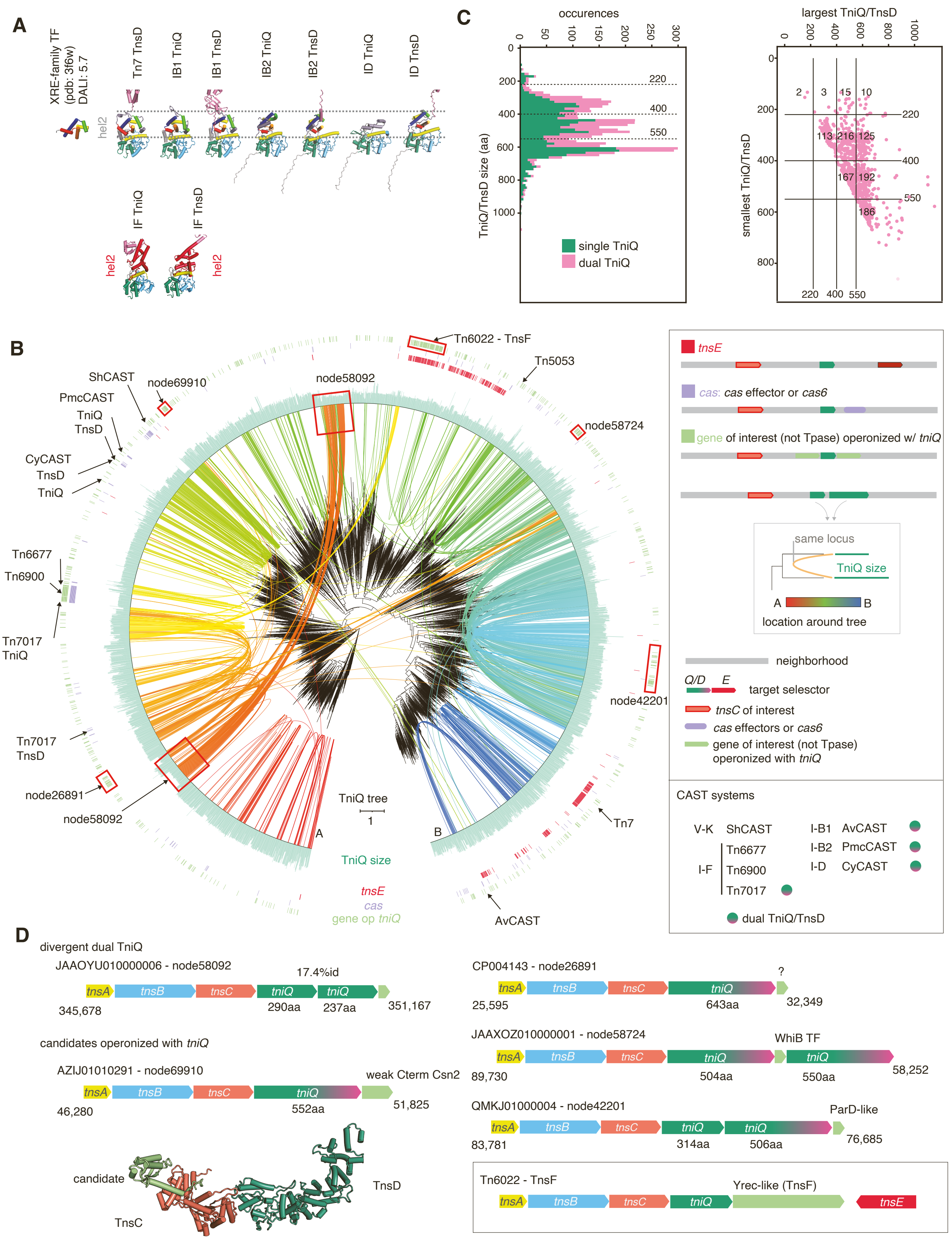
A
XRE-family TF (pdb: 3f6w) DALI: 5.7
Tn7 TnsD
IB1 TniQ
IB1 TnsD
IB2 TniQ
IB2 TnsD
ID TniQ
ID TnsD
hel2
IF TniQ
IF TnsD
hel2
hel2
C
occurences
TniQ/TnsD size (aa)
220
400
550
single TniQ
dual TniQ
largest TniQ/TnsD
smallest TniQ/TnsD
2
3
15
10
113
216
125
167
192
186
220
400
550
B
Tn6022 - TnsF
Tn5053
node58092
node69910
ShCAST
PmcCAST
TniQ
TnsD
CyCAST
TnsD
TniQ
node58724
Tn6677
Tn6900
Tn7017
TniQ
Tn7017
TnsD
node26891
node58092
node42201
Tn7
AvCAST
TniQ tree
1
A
B
TniQ size
tnsE
cas
gene op tniQ
tnsE
cas: cas effector or cas6
gene of interest (not Tpase) operonized w/ tniQ
same locus
TniQ size
A
B
location around tree
neighborhood
Q/D
E
target selecter
tnsC of interest
cas effectors or cas6
gene of interest (not Tpase) operonized with tniQ
CAST systems
V-K ShCAST
I-F Tn6677
Tn6900
Tn7017
I-B1 AvCAST
I-B2 PmcCAST
I-D CyCAST
dual TniQ/TnsD
D
divergent dual TniQ
JAAOYU010000006 - node58092
17.4%id
tnsA tnsB tnsC tniQ tniQ
345,678
290aa
237aa
351,167
candidates operonized with tniQ
AZIJ01010291 - node69910
weak Cterm Csn2
tnsA tnsB tnsC tniQ
46,280
552aa
51,825
candidate
TnsC
TnsD
CP004143 - node26891
?
tnsA tnsB tnsC tniQ
25,595
643aa
32,349
JAAXOZ010000001 - node58724
WhiB TF
tnsA tnsB tnsC tniQ tniQ
89,730
504aa
550aa
58,252
QMKJ01000004 - node42201
ParD-like
tnsA tnsB tnsC tniQ tniQ
83,781
314aa
506aa
76,685
Tn6022 - TnsF
Yrec-like (TnsF)
tnsA tnsB tnsC tniQ tnsE

Figure S2

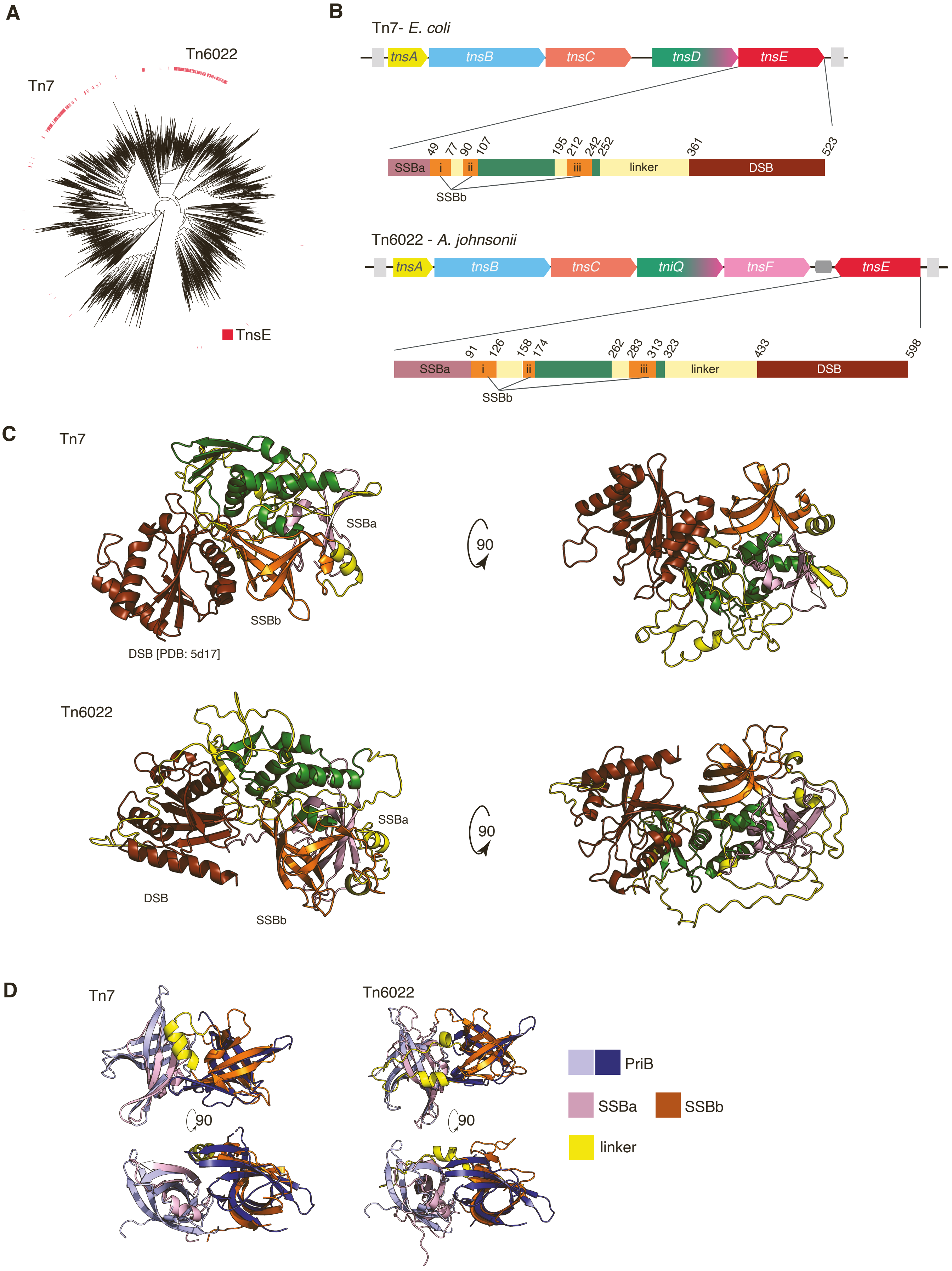
A
Tn7
Tn6022
TnsE
B
Tn7- E. coli
tnsA
tnsB
tnsC
tnsD
tnsE
49 77 90 107 195 212 242 252 361 523
SSBa
i
ii
iii
linker
DSB
SSBb
Tn6022 - A. johnsonii
tnsA
tnsB
tnsC
tniQ
tnsF
tnsE
91 126 158 174 262 283 313 323 433 598
SSBa
i
ii
iii
linker
DSB
SSBb
C
Tn7
SSBa
SSBb
DSB [PDB: 5d17]
90
Tn6022
SSBa
DSB
SSBb
90
D
Tn7
90
Tn6022
90
PriB
SSBa
SSBb
linker

Figure S3

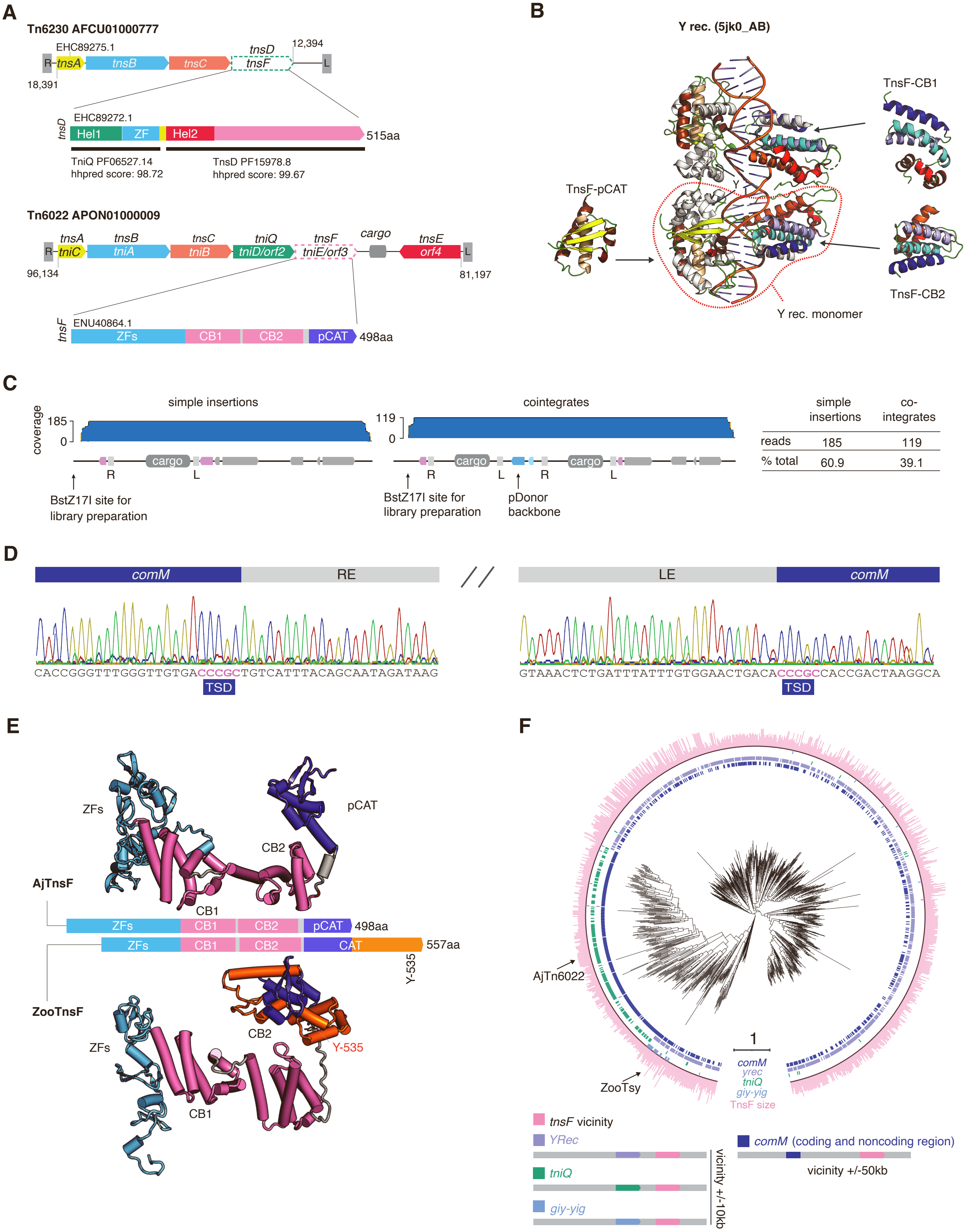

	simple insertions	co-integrates
reads	185	119
% total	60.9	39.1

Figure S4

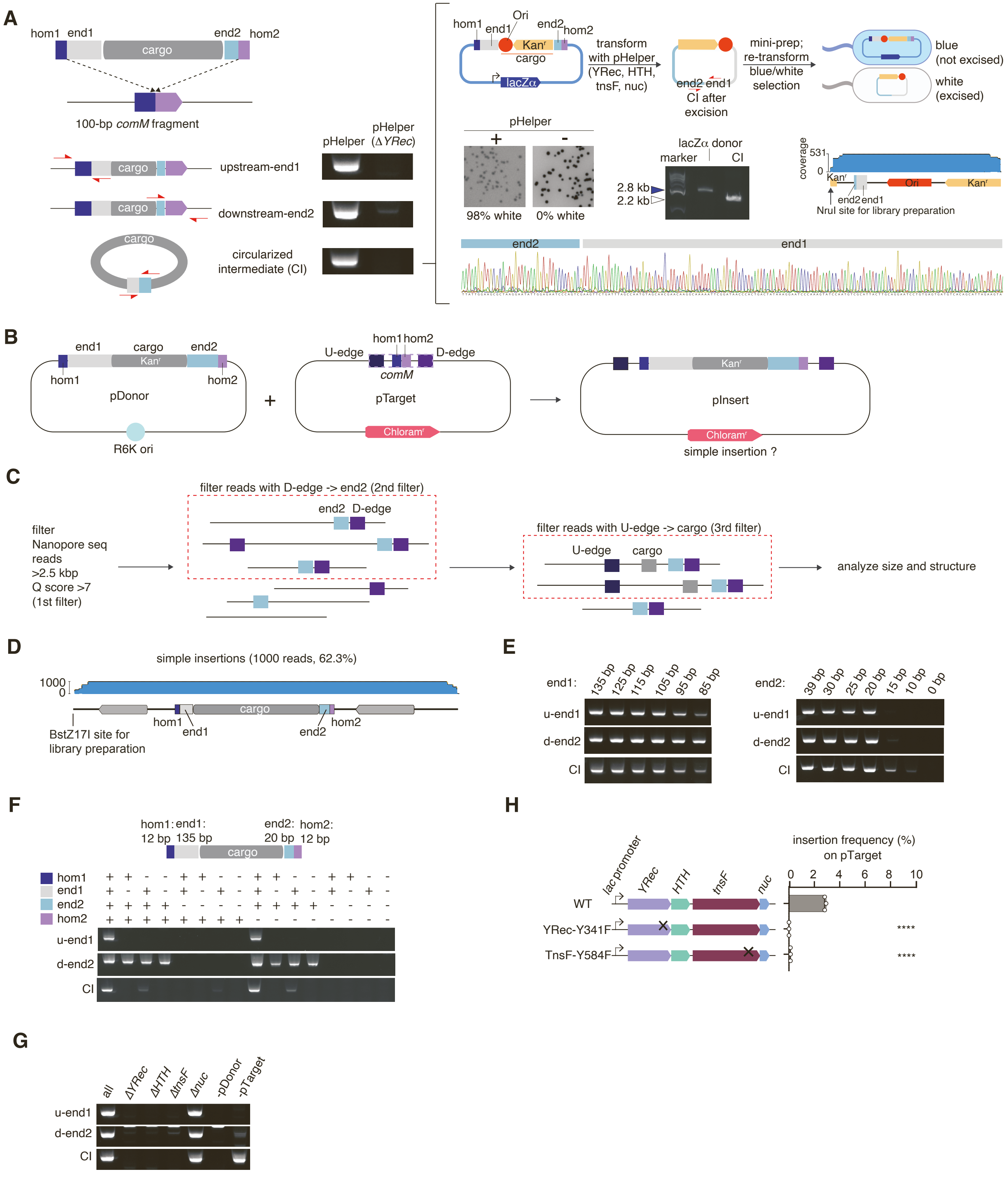

A
hom1 end1 cargo end2 hom2
100-bp comM fragment
pHelper
pHelper (ΔYRec)
upstream-end1
downstream-end2
circularized intermediate (CI)
Ori
Kanr
lacZα
transform with pHelper (YRec, HTH, tnsF, nuc)
end2 end1
CI after excision
mini-prep; re-transform blue/white selection
blue (not excised)
white (excised)
98% white
0% white
lacZα donor
marker
CI
2.8 kb
2.2 kb
coverage
531
0
NruI site for library preparation
B
pDonor
R6K ori
U-edge
D-edge
comM
pTarget
Chloramr
pInsert
simple insertion ?
C
filter Nanopore seq reads >2.5 kbp Q score >7 (1st filter)
filter reads with D-edge -> end2 (2nd filter)
end2 D-edge
filter reads with U-edge -> cargo (3rd filter)
U-edge cargo
analyze size and structure
D
simple insertions (1000 reads, 62.3%)
1000
0
BstZ17I site for library preparation
E
end1:
135 bp 125 bp 115 bp 105 bp 95 bp 85 bp
end2:
39 bp 30 bp 25 bp 20 bp 15 bp 10 bp 0 bp
u-end1
d-end2
CI
F
hom1: 12 bp
end1: 135 bp
end2: 20 bp
hom2: 12 bp
G
all ΔYRec ΔHTH ΔtnsF Δnuc -pDonor -pTarget
H
lac promoter
YRec HTH tnsF nuc
insertion frequency (%) on pTarget
0 2 4 6 8 10
WT
YRec-Y341F
TnsF-Y584F
****
****

Figure S5

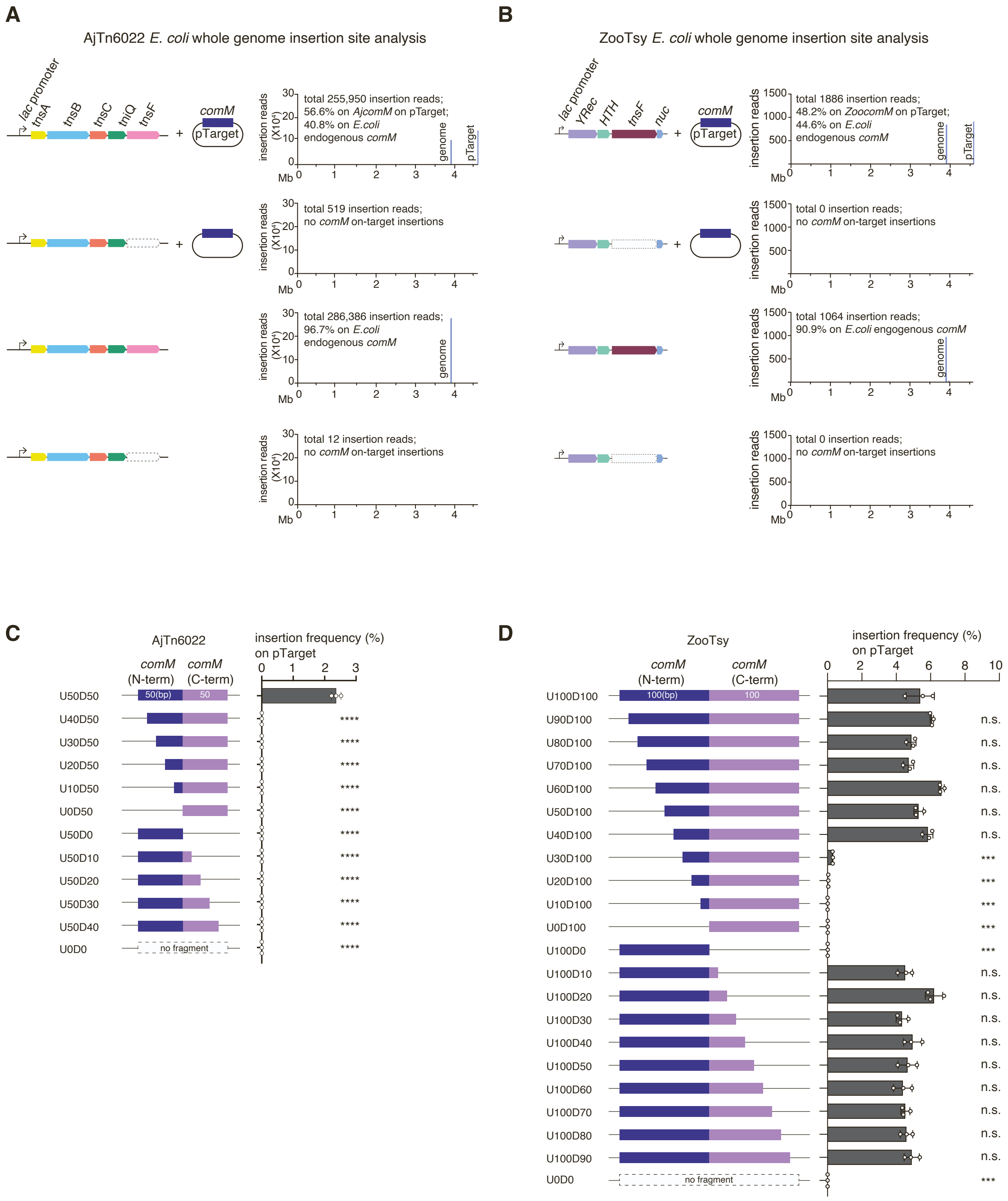
A
AjTn6022 E. coli whole genome insertion site analysis
lac promoter
tnsA
tnsB
tnsC
tniQ
tnsF
comM
pTarget
insertion reads (X10⁴)
Mb
genome
total 255,950 insertion reads;
56.6% on AjcomM on pTarget;
40.8% on E.coli endogenous comM
total 519 insertion reads;
no comM on-target insertions
total 286,386 insertion reads;
96.7% on E.coli endogenous comM
total 12 insertion reads;
no comM on-target insertions
B
ZooTsy E. coli whole genome insertion site analysis
lac promoter
YRec
HTH
tnsF
nuc
comM
pTarget
insertion reads
total 1886 insertion reads;
48.2% on ZoocomM on pTarget;
44.6% on E.coli endogenous comM
total 0 insertion reads;
no comM on-target insertions
total 1064 insertion reads;
90.9% on E.coli engogenous comM
total 0 insertion reads;
no comM on-target insertions
C
AjTn6022
comM (N-term)
comM (C-term)
insertion frequency (%) on pTarget
50(bp)
50
U50D50
U40D50
U30D50
U20D50
U10D50
U0D50
U50D0
U50D10
U50D20
U50D30
U50D40
U0D0
no fragment
****
D
ZooTsy
comM (N-term)
comM (C-term)
insertion frequency (%) on pTarget
100(bp)
100
U100D100
U90D100
U80D100
U70D100
U60D100
U50D100
U40D100
U30D100
U20D100
U10D100
U0D100
U100D0
U100D10
U100D20
U100D30
U100D40
U100D50
U100D60
U100D70
U100D80
U100D90
U0D0
no fragment
n.s.
***

Figures S6

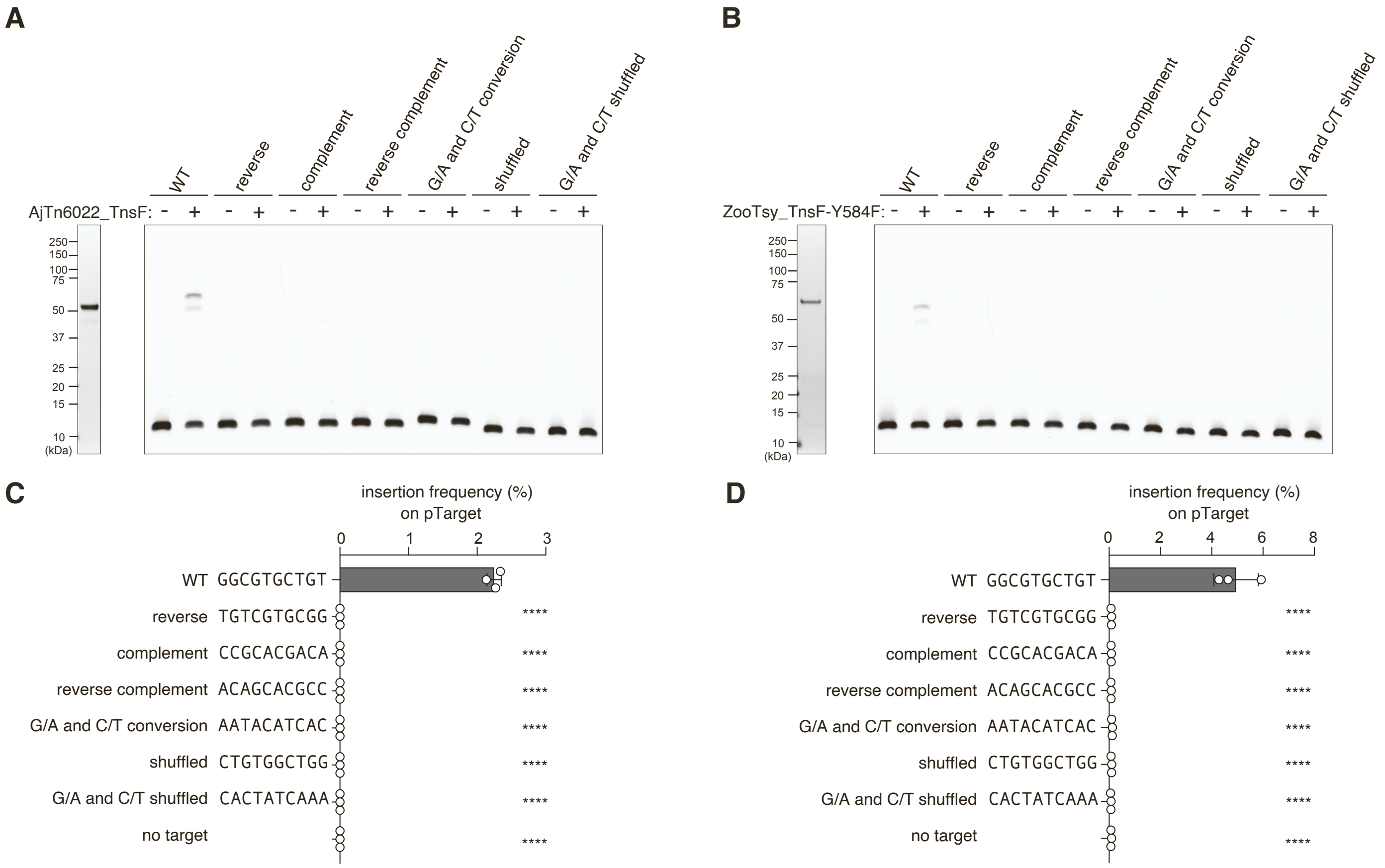
A
WT
reverse
complement
reverse complement
G/A and C/T conversion
shuffled
G/A and C/T shuffled
AjTn6022_TnsF:
250
150
100
75
50
37
25
20
15
10
(kDa)
B
ZooTsy_TnsF-Y584F:
C
insertion frequency (%)
on pTarget
0
1
2
3
WT GGCGTGCTGT
reverse TGTCGTGCGG
complement CCGCACGACA
reverse complement ACAGCACGCC
G/A and C/T conversion AATACATCAC
shuffled CTGTGGCTGG
G/A and C/T shuffled CACTATCAAA
no target
****
D
4
6
8

Figure S7
