# Supplementary figures and images for: Modularity and diversity of target selectors in Tn7 transposons
Source: Mol Cell. 2023 Jun 15;83(12):2122–2136.e10. doi: 10.1016/j.molcel.2023.05.013 (PMC10293859; doi:10.1016/j.molcel.2023.05.013)

**A**

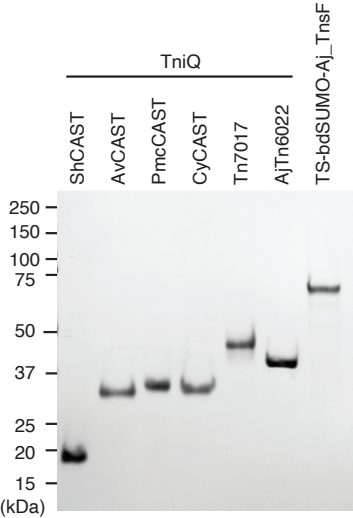

**B**

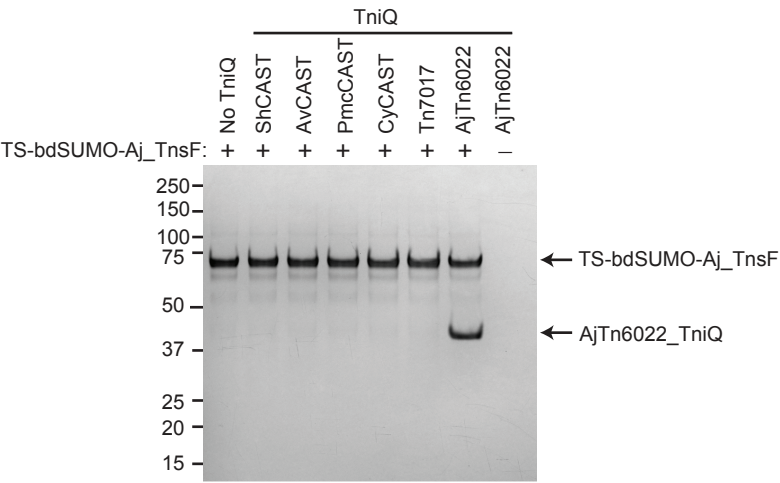

Supplement: Data S2. Raw gel images, related to Figure 5C — (A) Gel showing purified TniQ proteins. (B) Uncropped gel shown in Figure 5C. [file mmc3.pdf]
